# Increasing the bulk of the 1TEL–target linker and retaining the 10×His tag in a 1TEL–CMG2-vWa construct improves crystal order and diffraction limits

**DOI:** 10.1107/S2059798323007246

**Published:** 2023-09-25

**Authors:** Parag L. Gajjar, Maria J. Pedroza Romo, Celeste M. Litchfield, Miles Callahan, Nathan Redd, Supeshala Nawarathnage, Sara Soleimani, Jacob Averett, Elijah Wilson, Andrew Lewis, Cameron Stewart, Yi-Jie Tseng, Tzanko Doukov, Andrey Lebedev, James D. Moody

**Affiliations:** a Department of Chemistry and Biochemistry, Brigham Young University, 701 East University Parkway, Provo, UT 84602, USA; b Macromolecular Crystallography Group, Structural Molecular Biology Resource, Stanford Synchrotron Radiation Lightsource, Menlo Park, California, USA; c Scientific Computing, STFC Rutherford Appleton Laboratory, Didcot OX11 0QX, United Kingdom; University of Konstanz, Germany

**Keywords:** polymer-mediated protein crystallization, best practices, 1TEL, TELSAM, CMG2, ANTXR2, vWa domains, polymer flipping

## Abstract

TELSAM-fusion crystallization has the potential to become a revolutionary tool for the facile crystallization of proteins. TELSAM fusion can increase the crystallization rate and enable crystallization at low protein concentrations, in some cases with minimal crystal contacts [Nawarathnage *et al.* (2022 ▸), *Open Biol.* **12**, 210271]. Here, requirements for the linker composition between 1TEL and a fused CMG2 vWa domain were investigated. Ala-Ala, Ala-Val, Thr-Val and Thr-Thr linkers were evaluated, comparing metrics for crystallization propensity and crystal order. The effect on crystallization of removing or retaining the purification tag was then tested. It was discovered that increasing the linker bulk and retaining the 10×His purification tag improved the diffraction resolution, likely by decreasing the number of possible vWa-domain orientations in the crystal. Additionally, it was discovered that some vWa-domain binding modes are correlated with scrambling of the 1TEL polymer orientation in crystals and an effective mitigation strategy for this pathology is presented.

## Abstract 2

Using a 1TEL–CMG2-vWa construct, evidence is provided to support limiting the flexibility of linkers between TELSAM and proteins of interest and considering retaining polyhistidine purification tags in TELSAM-fusion constructs. The phenomenon of TELSAM-polymer flipping is also identified and a correction strategy is developed.

## 1. Introduction

Experimental structural characterization of proteins is essential for protein engineering, drug and target discovery, virtual drug screening and elucidation of protein structure–function relationships in health and disease (McFedries *et al.*, 2013 ▸; Maveyraud & Mourey, 2020 ▸). While cryo-electron microscopy is well suited for high-molecular-weight proteins, X-ray crystallography remains the tool of choice for obtaining high-resolution structures of lower molecular-weight proteins (Cooper *et al.*, 2011 ▸; Zhang & Liu, 2018 ▸). Since the introduction of X-ray crystallographic methods, approximately 5% of proteins investigated have resulted in a deposited structure. The protein crystallization process is generally laborious, time-consuming and expensive because of this high failure rate (Dale *et al.*, 2003 ▸; Kurgan & Mizianty, 2009 ▸; Terwilliger *et al.*, 2009 ▸). The recent advancement of micro-electron diffraction of protein microcrystals also supports the need for improved protein crystallization methods (Shi *et al.*, 2013 ▸). The continuous need for protein structure determination in various fields calls for a faster and more reliable technique for protein crystallization.

Protein crystallization chaperones can improve the success rate of protein crystallization. Currently available protein crystallization chaperone strategies can be divided into four main types: (i) noncovalent crystallization chaperones (for example nanobodies and DARPins; Koide, 2009 ▸; Kovari *et al.*, 1995 ▸), (ii) host–guest lattices (for example EngBF and R1EN; Ernst *et al.*, 2019 ▸; Maita, 2018 ▸), (iii) fused monomeric crystallization chaperones (for example glutathione *S*-transferase, maltose-binding protein, thioredoxin and green fluorescent protein; Uhlén *et al.*, 1992 ▸; Malhotra, 2009 ▸) and (iv) polymer-forming crystallization chaperones (for example TELSAM; Nauli *et al.*, 2007 ▸; Kim *et al.*, 2001 ▸).

The ‘TELSAM’ variant of the human translocation ETS leukaemia (TEL) protein sterile alpha motif (SAM) domain is a 75-amino-acid (9 kDa) polymer-forming crystallization chaperone that is soluble at pH > 8. However, when the pH decreases below 7, TELSAM forms a six-subunit-per-turn helical polymer that can enable fused target proteins to co-crystallize (Kim *et al.*, 2001 ▸; Nauli *et al.*, 2007 ▸; Nawarath­nage *et al.*, 2022 ▸). TELSAM reduces the entropic cost of crystal nucleation and growth by pre-freezing some degrees of freedom in the crystal lattice. Additionally, pre-ordering many thousands of copies of the target protein along the polymer results in substantial avidity to strengthen the subsequent target protein–target protein interactions that are formed when adjacent TELSAM polymers associate. As a result, TELSAM–target protein fusions readily form crystals. TELSAM is thus a compromise between host–guest lattices, which readily form crystals but as yet do not force the target protein to participate in crystal contacts, and fused monomeric crystallization chaperones, which force the target protein to participate in crystal contacts but are not guaranteed to form crystals (Nawarathnage *et al.*, 2022 ▸).

There are two principal strategies to fuse TELSAM and a protein of interest. The first employs a flexible linker (often polyglycine) that allows different possible orientations of the fused protein in the final crystal lattice, which may facilitate crystallization. The higher flexibility may alternatively lead to a greater degree of disorder in the crystal, leading to poorer diffraction resolution and higher *B* factors. The second strategy uses a rigid linker that imparts stability to the target-protein orientation to improve crystal order. The reduced linker flexibility may however block access to target-protein orientations optimal for crystallization, potentially reducing crystallization propensity or crystal quality (Nawarathnage *et al.*, 2022 ▸).

**Figure 1:**
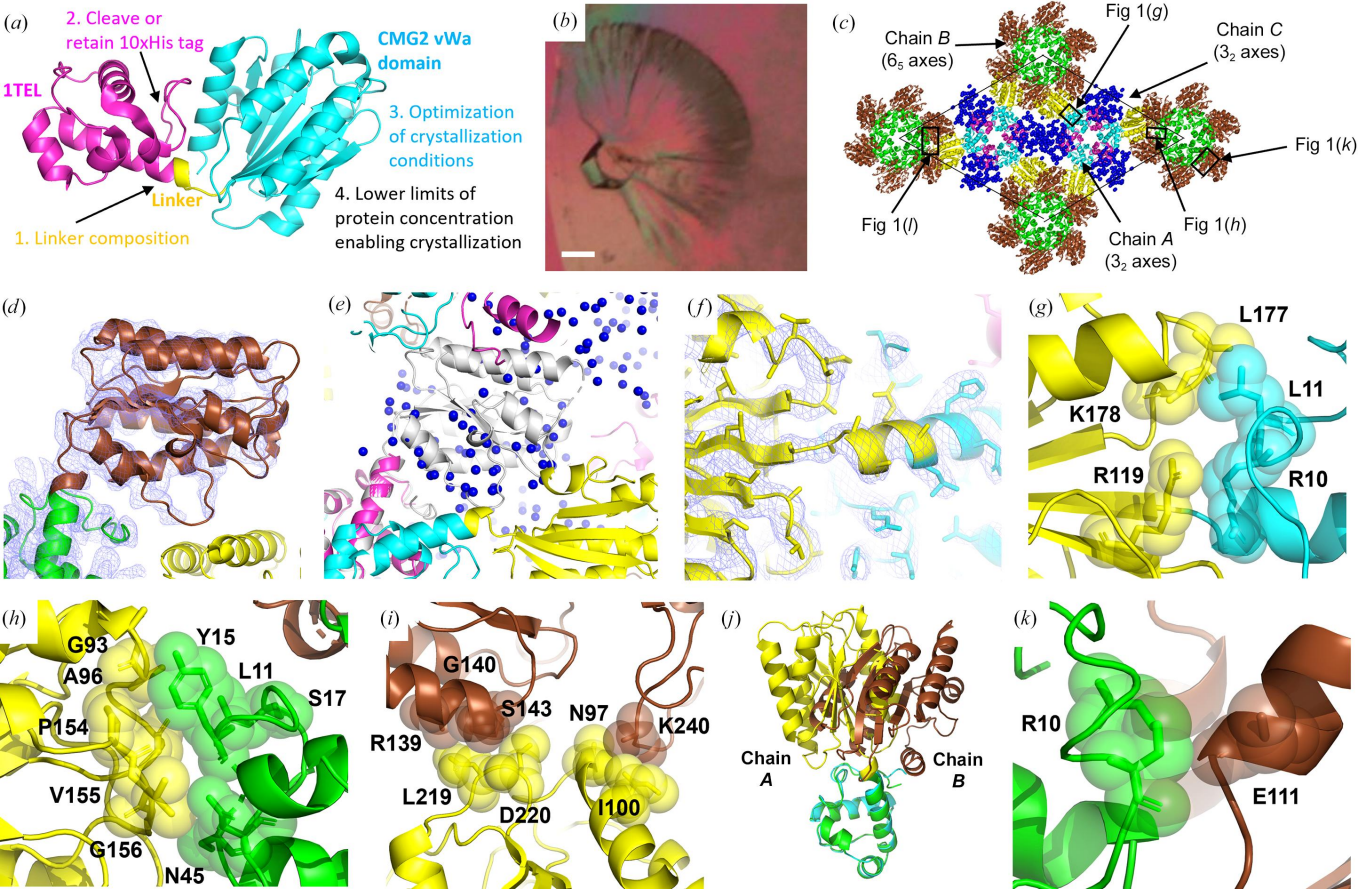
1TEL-AA-vWa (SUMO) crystal lattice. (*a*) Schematic of a 1TEL fusion indicating the variables investigated in this study. (*b*) Batch 2 1TEL-AA-vWa (SUMO) crystal fan. The scale bar is 100 µm. (*c*) Crystal lattice packing of the 1TEL-AA-vWa (SUMO) crystal, indicating the areas highlighted in subsequent panels. The TELSAM domains of chains *A*, *B* and *C* are shown in cyan, green and magenta, respectively, while the vWa domains of chains *A* and *B* are shown in yellow and brown, respectively. The unit cell is delineated by a thin black line. (*d*) Electron density (slate mesh) for the chain *B* vWa domain, contoured at 1.0σ. The 1TEL (green) and vWa (brown) domains are shown in cartoon representation for reference. (*e*) Detail of the region where the chain *C* vWa domain is expected to be found. A modelled orientation of the chain *C* vWa domain (white) is superimposed over the UNX atoms (blue spheres) used to delineate the unassigned electron density in this region. (*f*) Detail of the linker between the chain *A* 1TEL (cyan) and vWa domains (yellow). The electron density (slate mesh) is contoured at 1.0σ. (*g*) Detail of the interaction between the chain *A* vWa domain (yellow) and its host 1TEL domain (cyan). The proteins are shown in cartoon representation with selected amino acids shown as sticks and transparent spheres. (*h*) As in (*g*) but showing the interactions between the chain *A* vWa domain (yellow) and the chain *B* 1TEL domain (green). (*i*) As in (*g*) but showing the interactions between the chain *A* vWa domain (yellow) and two chain *B* vWa domains (brown). (*j*) Superposition of chain *A* and chain *B* 1TEL-AA-vWa chains via their 1TEL domains. (*k*) As in (*g*) but showing the chain *B* vWa (brown) and its host 1TEL (green).

In our previous study, we tested a 1TEL-flex-vWa construct consisting of residues 47–124 of human translocation ETS leukaemia [the sterile alpha motif (SAM) domain] followed by an alanine residue and then residues 40–217 of human capillary morphogenesis gene 2 (CMG2)/anthrax toxin receptor 2 (ANTXR2) [the von Willebrand factor type A (vWa) domain] (Fig. 1 ▸ *a*). This construct also featured an N-terminal 10×His-SUMO domain that was proteolytically removed during protein purification. We discovered that TELSAM fusion could increase the rate of crystallization, form crystals without direct contact between TELSAM polymers and enable crystallization at low protein concentrations, in some cases using only very minimal crystal contacts overall (Nawarathnage *et al.*, 2022 ▸). Replicating our previous results with 1TEL-flex-vWa uncovered potential areas for improvement of TELSAM-mediated protein crystallization.

(i) The linker length and sequence between TELSAM and the fusion protein dictates the available motion of the fused protein in the crystal. In the previous study, the 1TEL-flex-vWa linker consisted of a single alanine. In the resulting crystal lattice this alanine adopted an α-helical conformation, extending the C-terminal α-helix of 1TEL. The second and third amino acids of the vWa domain, Ala-Ala in this construct, preceded the first β-sheet of the vWa domain and adopted a more flexible conformation. No optimization of the 1TEL–vWa linker was carried out in this initial study.

(ii) A cleavable 10×His-SUMO tag was included in the construct to increase protein solubility and to allow His-tag removal before crystallization. The effects on crystallization propensity or crystal quality of SUMO-tag cleavage, SUMO protease enzyme activity or retaining the 10×His tag were not tested in the previous study.

(iii) In the previous study, we discovered that TELSAM can increase the crystallization rate but did not produce crystals that diffracted to better than 2.8 Å resolution. Optimization of the crystallization or cooling conditions was not investigated.

(iv) Although we determined that 1TEL-flex-vWa could be crystallized at concentrations as low as 1 mg ml^−1^, we did not determine the lower concentration limit for crystallization of this construct. Low-concentration protein crystallization using TELSAM is a promising avenue of investigation because it can potentially decrease batch sizes and the cost of protein crystallization, as well as enable the crystallization of poorly soluble and difficult-to-produce proteins.

As reproducibility in protein crystallization is desirable for drug screening and structure-based drug-design applications, we sought to explore these variables (Fig. 1 ▸ *a*) and address the issues identified.

## 2. Materials and methods

### 2.1. Cloning of 1TEL-AA-vWa (SUMO), 1TEL-AV-vWa (SUMO), 1TEL-TV-vWa (SUMO), 1TEL-TT-vWa (SUMO), 1TEL-AA-vWa and 1TEL-TV-vWa

The 1TEL-AA-vWa (formerly 1TEL-flex-vWa) construct from Nawarathnage *et al.* (2022 ▸) was used as the starting point for the design of the 1TEL–vWa variants used in this study. This construct placed residues 40–217 of the human anthrax toxin receptor 2 vWa domain (ANTXR2; also known as capillary morphogenesis gene 2, CMG2; UniProt P58335) downstream of residues 47–123 (the SAM domain) of human transcription factor ETV6 (ETS variant transcription factor 6, also known as translocation ETS leukaemia protein, TEL; UniProt P41212). The pH-sensitive variant of this SAM domain is hereafter referred to as 1TEL. An alanine linker was placed between the two domains and substitutions relative to the wild-type proteins included R49A, V112E, K122A, R41A and C175A (Nawarathnage *et al.*, 2022 ▸). The AV, TV and TT substitutions were designed at positions 41 and 42 of the vWa domain using *PyMOL* (version 2.0; Schrödinger) and *Geneious* (version 9.1.8; https://www.geneious.com). Gene fragments were synthesized by Twist Bioscience (https://www.twistbioscience.com) and were assembled into the pET42_SUMO vector (Walls *et al.*, 2022 ▸; cut with XhoI) using Gibson assembly (Gibson *et al.*, 2009 ▸) to generate 1TEL-AV-vWa (SUMO), 1TEL-TV-vWa (SUMO) and 1TEL-TT-vWa (SUMO) constructs. 10×His-1TEL-AA-vWa and 10×His-1TEL-TV-vWa were cloned in the same manner, being assembled into a pET42_SUMO vector that was first cut with XhoI and NdeI to remove the SUMO domain. The sequence MGHHHHHHHHHH was directly appended to the N-terminus of 1TEL (SIRL…) in these SUMO tag-free constructs. All constructs were introduced into *Escherichia coli* BL21(DE3) cells and sequence-verified by Sanger sequencing in both directions by Eton Bioscience (https://www.etonbio.com).

### 2.2. Protein expression

20 ml lysogeny broth (LB) medium with 100 µg ml^−1^ kanamycin was inoculated with a stab of frozen cell stock and shaken at 37°C and 250 rev min^−1^ overnight. The following day, 15 ml of the overnight culture was added to 1 l LB supplemented with 0.05% glucose and 100 µg ml^−1^ kanamycin and incubated at 37°C and 250 rev min^−1^ until the optical density (OD) reached 0.6. At an OD of 0.6, isopropyl β-d-1-thiogalactopyranoside (IPTG) was added to a final concentration of 100 µ*M*. The culture was incubated at 18°C and 200 rev min^−1^ overnight. The following day, the cells were collected by centrifugation, snap-frozen in liquid nitrogen and stored at −80°C.

### 2.3. Purification of 1TEL-AA-vWa (SUMO), 1TEL-AV-vWa (SUMO), 1TEL-TV-vWa (SUMO) and 1TEL-TT-vWa (SUMO)

All purification steps were performed on ice or in a refrigerator at 4°C. Cell pellets (20 g) were resuspended in a fivefold excess of wash buffer (50 m*M* Tris, 200 m*M* KCl, 50 m*M* imidazole pH 8.8) containing 1 m*M* phenylmethyl­sulfonyl fluoride (PMSF), 100 µ*M* dithiothreitol (DTT), 0.5 mg ml^−1^ lysozyme and 800 n*M* deoxyribonuclease I. The suspended cells were then sonicated at 60% power at 12 s on/59 s off for 25 cycles (Qsonica Q500) in a spinning ice bath. The resulting lysate was centrifuged at 40 000*g* and the supernatant was loaded onto 6 ml HisPure Ni–NTA resin (Thermo Scientific). The column was then washed with 7 column bed volumes (CV) of wash buffer (50 m*M* Tris pH 8.8, 200 m*M* KCl, 50 m*M* imidazole). The protein was then eluted with about 7 CV of elution buffer (50 m*M* Tris pH 8.8, 200 m*M* KCl, 400 m*M* imidazole) until protein stopped appearing in subsequent fractions as detected using Bradford reagent (Bradford, 1976 ▸). The collected protein was then desalted using several PD-10 desalting columns in parallel (Cytiva). The SUMO tag was removed by incubating the protein overnight at 4°C with 0.5 mg SUMO protease (Lau *et al.*, 2018 ▸) per 100 ml protein. The SUMO protease and cleaved SUMO tags were removed by flowing the cleavage reaction over 2 ml fresh Ni–NTA resin. The protein was then concentrated to 3 ml and diluted tenfold with water (to decrease the conductivity to below 3 mS cm^−1^). The diluted protein was loaded onto 5 ml Source 15Q anion-exchange resin (Cytiva) and eluted with 50 m*M* Tris pH 8.8, 1 *M* KCl using gradient elution. Fractions containing the pure protein of interest were concentrated to 2 ml and loaded onto a 100 ml Superdex 200 prep-grade size-exclusion column (Cytiva). The proteins were judged to be greater than 95% pure by SDS–PAGE.

### 2.4. Purification of 1TEL-AA-vWa, 1TEL-TV-vWa and vWa alone

These proteins were purified in an identical manner to their SUMO-tagged counterparts above, except that the SUMO tag cleavage, tag removal and ion-exchange chromatography steps were omitted.

### 2.5. Crystallization and diffraction of 1TEL-AA-vWa (SUMO), 1TEL-AV-vWa (SUMO), 1TEL-TV-vWa (SUMO), 1TEL-TT-vWa (SUMO), His-1TEL-AA-vWa, His-1TEL-TV-vWa and vWa alone

The concentration of the purified protein was adjusted to 1 and 20 mg ml^−1^. His-1TEL-TV-vWa was additionally adjusted to 0.1, 0.2, 0.5, 1, 2, 5, 10, 15 and 20 mg ml^−1^ for the protein-concentration trial. The concentration of vWa alone was only adjusted to 20 mg ml^−1^. Commercially available crystallization screens (PEG Ion, Salt RX and Index from Hampton Research) and custom screens (PEG-custom and ammonium sulfate) were screened at the above protein concentrations. 1.2 µl of the indicated protein solutions was combined with 1.2 µl reservoir solution (SPT Labtech Mosquito) and equilibrated against 50 µl reservoir solution in a sitting-drop vapour-diffusion format. Crystals appeared after two days for His-1TEL-TV-vWa, 3–7 days for 1TEL-AA-vWa (SUMO), 1TEL-AV-vWa (SUMO), 1TEL-TV-vWa (SUMO) and His-1TEL-AA-vWa, and 35 days for vWa alone. No crystals appeared for 1TEL-TT-vWa (SUMO).

The largest 1TEL-AA-vWa (SUMO) crystals appeared in 100 m*M* sodium acetate pH 4.6, 2.0 *M* sodium formate. Other conditions giving 1TEL-AA-vWa (SUMO) crystals included (i) 100 m*M* bis-Tris pH 5.7, 3.0 *M* NaCl (the same condition as gave crystals in our previous study; Nawarathnage *et al.*, 2022 ▸); (ii) 0.2 *M* ammonium sulfate, 0.1 *M* HEPES pH 7.5, 25%(*w*/*v*) polyethylene glycol 3350; (iii) 100 m*M* Tris pH 8.5, 200 m*M* ammonium acetate, 25%(*w*/*v*) polyethylene glycol 3350; (iv) 100 m*M* Tris pH 8.5, 200 m*M* ammonium sulfate, 25%(*w*/*v*) polyethylene glycol 3350; (v) 100 m*M* bis-Tris pH 5.5, 200 m*M* ammonium acetate, 25%(*w*/*v*) polyethylene glycol 3350; (vi) 1.8 *M* ammonium citrate pH 7.0; (vii) 200 m*M* ammonium formate pH 6.6, 20%(*w*/*v*) polyethylene glycol 3350; (viii) 200 m*M* ammonium acetate pH 7.1, 20%(*w*/*v*) polyethylene glycol 3350; (ix) 100 m*M* bis-Tris pH 6.25, 130 m*M* magnesium formate and (x) 40 m*M* sodium phosphate, 960 m*M* potassium phosphate pH 8.2. Crystals also appeared in many conditions containing just polyethylene glycol 3350 at concentrations between 16% and 24% at pH values of 6.5, 8.5 and 9.0 and with MES, Tris and glycine buffers.

The largest His-1TEL-AA-vWa crystals appeared in 100 m*M* bis-Tris pH 5.7, 3.0 *M* NaCl and in 0.07 *M* bis-Tris propane pH 7.8, 0.03 *M* citric acid, 20% PEG 3350. The largest 1TEL-AV-vWa (SUMO), 1TEL-TV-vWa (SUMO) and His-1TEL-TV-vWa crystals appeared in 1.3–2.0 *M* ammonium sulfate, 0.1 *M* HEPES pH 5–8.5. Crystals were cryoprotected by briefly passing them through a solution of 20% glycerol in reservoir solution prior to cooling in liquid nitrogen.

### 2.6. Data collection, reduction and structure solution

X-ray diffraction data were collected remotely on SSRL beamlines 9-2, 12-1 and 12-2. Diffraction data for most crystals were obtained at an energy of 12 658 eV. The data for 1TEL-AV-vWa (SUMO) and selected 1TEL-TV-vWa (SUMO) crystals were obtained at an energy of 12 398 eV. Diffraction data for iodine-treated crystals were obtained at an energy of 6690 eV. Typically, 120–360° of data were collected in 0.1° or 0.2° increments with 0.2 s exposures.

The *autoPROC* pipeline (Vonrhein *et al.*, 2011 ▸), encompassing the *XDS* algorithm (Kabsch, 2010 ▸), was used to process the data sets, and molecular replacement using *Phenix Phaser* (Liebschner *et al.*, 2019 ▸; McCoy *et al.*, 2007 ▸) was employed to solve the phases. Structure rebuilding was performed in *Coot *(Emsley *et al.*, 2010 ▸) and refinement in *phenix.refine* (Afonine *et al.*, 2012 ▸). TLS parameters were refined using TLS groups determined by *Phenix*. Refinement was assisted using statistics from the *MolProbity* server (Chen *et al.*, 2010 ▸; Williams *et al.*, 2018 ▸).

The diffraction images of the 1TEL-TV-vWa (SUMO) crystal were processed with *DIALS* (Winter *et al.*, 2018 ▸) using the global background model (Parkhurst *et al.*, 2016 ▸). The integrated data were scaled and merged using *AIMLESS* (Evans & Murshudov, 2013 ▸). The complete data set of intensities was split into two data sets with *h* − *k* = 3*n* and *h* − *k* = 3*n* ± 1 using a purpose-made script written in *R* (https://www.r-project.org). The *I*-to-*F* conversion was completed separately for the two partial data sets using *CTRUNCATE* from the *CCP*4 suite (Winn *et al.*, 2011 ▸). The initial model was obtained using the *h* − *k* = 3*n* data set and *MOLREP* (Vagin & Teplyakov, 2010 ▸). The *h* − *k* = 3*n* and *h* − *k* = 3*n* ± 1 data sets were separately rescaled to *F*_calc_ using *REFMAC* (Murshudov *et al.*, 2011 ▸) with zero cycles of coordinate refinement and joined using an *R* script. The atomic model was then refined against the joined data set using *REFMAC*, with the procedure involving separate scaling and joining. Model refinement was repeated four times, alternated with sessions of model inspection and correction with *Coot* (Emsley *et al.*, 2010 ▸). To evaluate the structure-amplitude correction that has in effect been applied to the *h* − *k* = 3*n* ± 1 data set, the final *h* − *k* = 3*n* and *h* − *k* = 3*n* ± 1 data sets were fitted separately to the complete set of structure amplitudes generated from the complete set of merged intensities using *CTRUNCATE*. The fitting was completed using an *R* script and the relative values of the overall scale factor [2.658 (2)], *B*_11_ [5.18 (3) Å^2^] and *B*_33_ [2.13 (4) Å^2^] were calculated from the respective absolute values. This translates into a relative scaling coefficient of about 7 in terms of intensities.

## 3. Results

### 3.1. 1TEL-AA-vWa (SUMO) does not reliably form well diffracting crystals

The first batch of 1TEL-flex-vWa [hereafter termed 1TEL-AA-vWa (SUMO)] generated large hexagonal crystals in three days in a single condition (100 m*M* bis-Tris pH 5.7, 3.0 *M* NaCl) with an average diffraction limit of 2.9 Å (and a range of 2.8–3.0 Å), as reported previously (Nawarathnage *et al.*, 2022 ▸).

After having successfully crystallized the human CMG2 vWa domain via fusion to 1TEL (Nawarathnage *et al.*, 2022 ▸), we sought to determine the binding mode of a therapeutic drug candidate, PGM (1,2,3,4,6-penta-*O*-galloyl-β-d-mannopyranose; Doyaguez *et al.*, 2019 ▸), to the vWa domain. A different team of students prepared two new batches (batches 2 and 3) of 1TEL-AA-vWa (SUMO). Rather than focusing our crystallization screen only on those conditions that gave large crystals from batch 1, we screened subsequent batches of 1TEL-AA-vWa (SUMO) against a broad variety of 288 sparse-matrix commercial conditions and 96 custom conditions. We have observed that TELSAM fusion does not appear to remove the need to screen a broad variety of crystallization conditions, as different constructs give crystals under widely varying conditions.

Both batches 2 and 3 of 1TEL-AA-vWa (SUMO) yielded crystals in around ten days, but with a different crystal habit (needles occurring separately or in a fan; Fig. 1 ▸ *b*) and greater propensity (nine new crystallization conditions; Section 2) than batch 1. A total of 43 crystals spanning each of these new conditions were selected for X-ray diffraction studies. Some crystals from each batch were soaked with 1 m*M* PGM (in 1% DMSO) for 16 h prior to mounting and cooling. Only three of these soaked crystals diffracted to better than 4 Å resolution. A portion of the protein from each batch was instead co-crystallized with PGM. This proved challenging because PGM rapidly crystallized on its own and often prevented protein crystal formation. Eight crystals obtained from co-crystallization experiments were screened for X-ray diffraction, but none diffracted X-rays.

To determine the robustness and reproducibility of TELSAM-mediated crystallization, a third team of students prepared two additional batches of 1TEL-AA-vWa (SUMO). A fourth batch of protein produced crystals in a single condition (40 m*M* sodium phosphate monobasic, 960 m*M* potassium phosphate pH 8.2) distinct from those identified previously. These crystals were not soaked or co-crystallized with PGM and did not diffract X-rays. A fifth batch did not form crystals.

Batch 2 1TEL-AA-vWa crystals featured target proteins in multiple orientations relative to their host 1TEL domains across chains in the asymmetric unit, in multiple orientations within a single chain or that were not located. The batch 2 crystals that diffracted X-rays were obtained in 100 m*M* sodium acetate pH 4.6, 2.0 *M* sodium formate. One of these crystals diffracted to 3.3 Å resolution (Table 1 ▸). Molecular replacement with this 1TEL-AA-vWa (SUMO) crystal was carried out using PDB entries 2qar (1TEL) and 1sht (vWa) as search models, yielding a solution with 1TEL polymers along the 6_5_ axes and two additional 1TEL polymers along the two 3_2_ axes of the *P*6_5_ unit cell (Fig. 1 ▸ *c*). The asymmetric unit thus contains one 1TEL domain (chain *B*) from a polymer lying on a 6_5_ axis and two 1TEL domains (chains *A* and *C*) from a polymer lying along a 3_2_ axis. The vWa domains corresponding to the chain *A* and *B* 1TEL domains were also located, but the density for the 6_5_ axis vWa domain (chain *B*) was incomplete, suggesting that this chain *B* vWa domain may exist in at least two orientations relative to its host 1TEL domain (Fig. 1 ▸ *d*). The vWa domain corresponding to the chain *C* 1TEL domain (3_2_ axis) in the asymmetric unit was not located, although there is ample space in the unit cell for it and ample unassigned electron density in the area where it is expected to be found. We began by modelling extended polyalanine peptides into this unassigned electron density and later replacing these with UNX atoms modelled at the coordinates of the peptide C^α^ atoms to approximate the area occupied by the various putative chain *C* vWa orientations (Fig. 1 ▸ *e*).

These observations suggest that the chain *C* vWa domain may exist in a variety of orientations relative to its host 1TEL polymer. Taken together, these data suggest that the vWa domains in this crystal may adopt at least six different orientations (one in chain *A*, at least two in chain *B* and at least three in chain *C*). An alternative possibility is that interaction with the PGM drug molecule may have partially misfolded the chain *B* and *C* vWa domains or disordered their positions in the crystal. In this scenario, the chain *B* vWa domain may still be partially resolved because it interacts with two copies of chain *A* vWa domains from two distinct 3_2_-axis polymers. This second possibility is supported by the fact that PGM soaking invariably worsened the diffraction quality of 1TEL–vWa crystals relative to crystals that had not been soaked with PGM. However, no clear sign of PGM was detected in the electron density.

We were able to refine this model to only relatively poor *R*_work_ and *R*_free_ values of 0.28 and 0.31, respectively, which diminishes confidence in the space-group assignment and molecular-replacement solution. Fortunately, TELSAM-fusion crystal structures have two built-in sources of validation which bolster confidence in this space-group assignment and solution: (i) the 1TEL monomers form the expected monomer–monomer interfaces and sixfold left-handed helical polymer and (ii) the N-termini of the vWa domains are within the expected distance of the C-termini of their host 1TEL monomers and continuous electron density can be seen for the 1TEL–vWa linkers (Fig. 1 ▸ *f*). This is notable because molecular replacement placed the 1TEL and vWa domain search models as independent monomers and included no constraints to enforce the observed symmetry or contacts. No twinning or pseudosymmetry was detected in this crystal.

We note that the well resolved chain *A* vWa domain (hosted by a 1TEL domain in the 3_2_-axis polymer) adopts a novel binding mode to its host 1TEL polymer. Considering its evidently critical role in maintaining the integrity of the crystal, the chain *A* vWa domain makes surprisingly minimal contact with its host 1TEL domain, burying only 270 Å^2^ of solvent-accessible surface area (average of both sides of the interface) in a fairly solvated interface. Specifically, Arg119, Leu177 and Lys178 of the vWa domain make van der Waals contacts with Arg10 and Leu11 of 1TEL (Fig. 1 ▸ *g*).

The chain *A* vWa domain also makes direct contacts to the chain *B* 1TEL and vWa domains (in the 6_5_-axes polymer), allowing the structural integrity of this crystal to be maintained (Fig. 1 ▸ *c*). These interactions are distinct from those observed in the previous batch 1 structure of 1TEL-AA-vWa (SUMO) (PDB entry 7n1o; Nawarathnage *et al.*, 2022 ▸). The chain *A* interface with the chain *B* 1TEL domain buries 252 Å^2^ of solvent-accessible surface area and is largely hydrophobic. Specifically, the side and main chains of Gly93, Ala96, Asn97, Pro154, Val155 and Gly156 in the vWa domain form hydrophobic and van der Waals contacts with the side and main chains of Leu11, Tyr15, Ser17 and Asn45 of the chain *B* 1TEL (Fig. 1 ▸ *h*). The interface of the chain *A* vWa domain with the chain *B* vWa domain buries 99 Å^2^ of solvent-accessible surface area and is more polar. Specifically, the side and main chains of Leu219 and Asp220 of the chain *A* vWa domain make hydrophobic and van der Waals interactions with Arg139, Gly140 and Ser143 of the chain *B* vWa domain (Fig. 1 ▸ *i*). This chain *A* vWa domain also forms contacts with a second chain *B* vWa domain from the same polymer as the first, burying 164 Å^2^ of solvent-accessible surface area. Specifically, Asn97 and Ile100 of the chain *A* vWa domain make van der Waals interactions with Lys240 from this second chain *B* vWa domain (Fig. 1 ▸ *i*).

The resolvable portions of the chain *B* vWa domain reveal that it adopts a binding mode to its host 1TEL polymer that is distinct from that of the chain *A* vWa domain but is likely to be similar to that of the unresolved chain *C* vWa domain (Fig. 1 ▸ *j*). The chain *B* vWa domain makes only a glancing contact with its host 1TEL polymer, involving van der Waals contacts between Glu111 of the vWa domain and Arg10 of the 1TEL domain and burying 120 Å^2^ of solvent-accessible surface area (Fig. 1 ▸ *k*). This minimal contact may explain why the chain *B* vWa domain may exist in more than one orientation relative to its host 1TEL domain.

As the three vWa domains in the asymmetric unit appear to adopt at least six binding modes (clustered around two general binding modes; Fig. 1 ▸ *i*), this batch 2 1TEL-AA-vWa (SUMO) structure suggests that fused target proteins may be able to choose their binding modes to their host TELSAM polymers at the time of polymer–polymer association. Further evidence comes from the extremely weak interactions that the chain *A* and *B* vWa domains make with their host 1TEL polymers. It is unlikely that these weak interactions would be made maintained prior to or in the absence of polymer–polymer association. This conclusion should be treated with caution since PGM treatment offers a possible alternative explanation for the disordered vWa domains in this crystal. Nevertheless, the multiple observed binding modes in this crystal establish that the vWa domain can stably adopt multiple binding modes and that doing so does not abolish useful X-ray diffraction.

### 3.2. Decreasing the flexibility of the 1TEL–vWa linker improved the diffraction resolution but did not improve the crystal quality

In the batch 2 1TEL-AA-vWa (SUMO) crystal described in the previous sections, we discovered the vWA domains in at least six different orientations among the three chains of the asymmetric unit, suggesting that the vWa domain may not readily find a single lowest-energy binding mode against its host 1TEL polymer during polymer–polymer association and that while failure to do so complicates structure solution, it apparently does not necessarily abrogate the formation of diffracting crystals. We hypothesized that for TELSAM–target-protein fusions to form well diffracting crystals with well resolved target proteins, all of the fused target proteins must find the same binding mode to their host polymers so as to allow the TELSAM–target-protein polymers to reproducibly find a single binding mode to one another. We further hypothesized that modification of the 1TEL–vWa linker could limit the orientational flexibility of the vWa domain such that all of the vWa domains in a crystal would more easily adopt the same binding mode against their host 1TEL polymers, thus being resolved in the resulting electron density and potentially increasing the diffraction resolution of the crystal.

**Figure 2:**
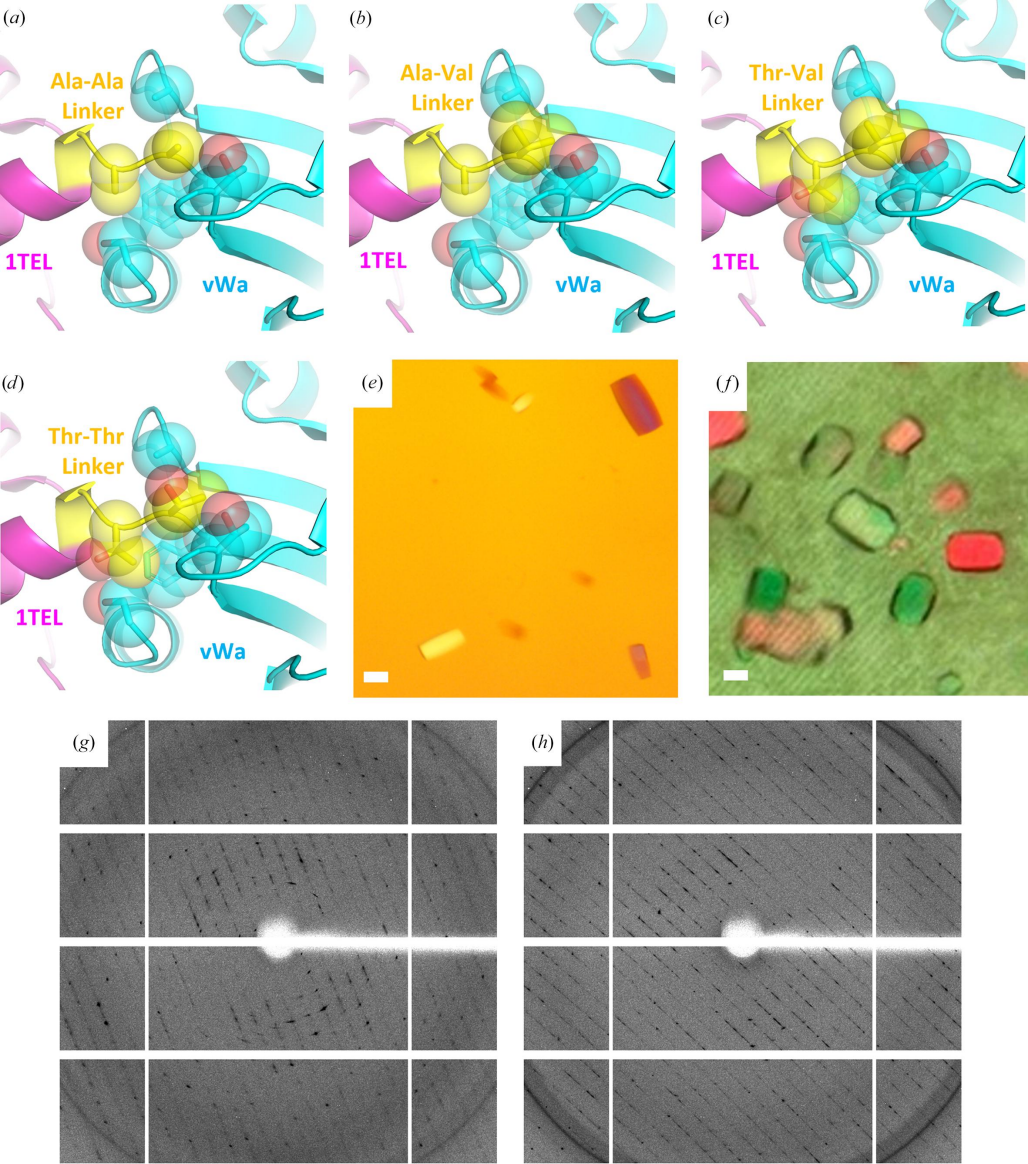
The results of adding bulk to the 1TEL–vWa linker. (*a*) Linker detail of the 1TEL-AA-vWa (SUMO) structure (PDB entry 7n1o) with 1TEL in magenta, the Ala-Ala linker in yellow and vWa in cyan. The linker and surrounding amino acids are shown as sticks and transparent spheres. (*b*) As in (*a*) but for the design model of 1TEL-AV-vWa (SUMO). (*c*) As in (*a*) but for the design model of 1TEL-TV-vWa (SUMO). (*d*) As in (*a*) but for the design model of 1TEL-TT-vWa (SUMO). (*e*) Representative crystals of 1TEL-TV-vWa (SUMO). The scale bar is 100 µm. (*f*) As in (*e*) but for 1TEL-AV-vWa (SUMO). (*g*) Representative diffraction pattern of a 1TEL-TV-vWa (SUMO) crystal. (*h*) As in (*g*) but for a 1TEL-AV-vWa (SUMO) crystal.

The single alanine linker used in the previous 1TEL-AA-vWa (SUMO) structure (PDB entry 7n1o) became part of the rigid C-terminal α-helix of the 1TEL domain. The second and third amino acids of the vWa domain (Ala-Ala in this construct) were seen in an extended conformation and did not pack well against the vWa-domain amino acids around them, suggesting that they are the most flexible part of the 1TEL–vWa connection in this crystal, despite lying within the vWa domain rather than in the designed linker (Fig. 2 ▸ *a*; Nawarathnage *et al.*, 2022 ▸).

We substituted the Ala-Ala linker with either Ala-Val (AV), Thr-Val (TV) or Thr-Thr (TT). These substitutions packed better with the amino acids around them and were intended to confer rigidity to the 1TEL–vWa connection (Figs. 2 ▸ *b*–2 ▸ *d*). We cloned the three different linker variants to create 1TEL-AV-vWa (SUMO), 1TEL-TV-vWa (SUMO) and 1TEL-TT-vWa (SUMO) constructs. We expressed, purified and crystallized each construct at both 1 and 20 mg ml^−1^ with a range of commercially available and custom-made crystallization screens. We executed a single round of optimization of the crystallization conditions, but no optimization of cryoprotection or cooling conditions.

1TEL-AV-vWa (SUMO) and 1TEL-TV-vWa (SUMO) each crystallized in four different conditions in 2–3 days, while 1TEL-TT-vWa (SUMO) did not crystallize (Figs. 2 ▸ *e* and 2 ▸ *f*). We identified initial hits in commercial crystallization screens, and from these hits we developed an ammonium sulfate optimization screen. 1TEL-AV-vWa (SUMO) crystals diffracted to an average resolution of 2.9 Å [*I*/σ(*I*) ≥ 2.0 at 2.5–3.5 Å across eight crystals]. For these crystals we were able to index an average of 35% of non-ice reflections (range 12–73%), with an average mosaicity of 0.59° (range 0.23–1.1°) and an average ISa of 20 (range 6–36) (Diederichs, 2010 ▸; Table 1 ▸). 1TEL-TV-vWa (SUMO) crystals diffracted to an average resolution of 2.7 Å [*I*/σ(*I*) ≥ 2.0 at 2.3–3.0 Å across six crystals]. For these crystals, we were able to index and average 49% of non-ice reflections (range 15–88%), with an average mosaicity of 0.55° (range 0.18–1.1°) and an average ISa of 19 (range 7–34). ISa [*I*/σ(*I*)^asymptotic^] is a measure of the intrinsic error in diffraction data collection, which is dependent on both crystal quality and the experimental setup. Excellent crystals and setups have ISa values greater than 40, while data with ISa values of at least 15–20 are still useful. Data with ISa values below 10 are considered poor (Diederichs, 2010 ▸).

By comparison, the batch 1 1TEL-AA-vWa (SUMO) crystals gave an average resolution of 2.9 Å (range 2.8–3.0 Å across three crystals), indexed 57% of non-ice reflections (range 43–67%), exhibited an average mosaicity of 0.47° (range 0.38–0.60°) and had an average ISa of 16 (range 15–17) (Nawarathnage *et al.*, 2022 ▸). These results suggest that the Thr-Val linker performed slightly better than the Ala-Ala linker, which in turn performed on a par with the Ala-Val linker, while the Thr-Thr linker abrogated crystal formation.

**Figure 3:**
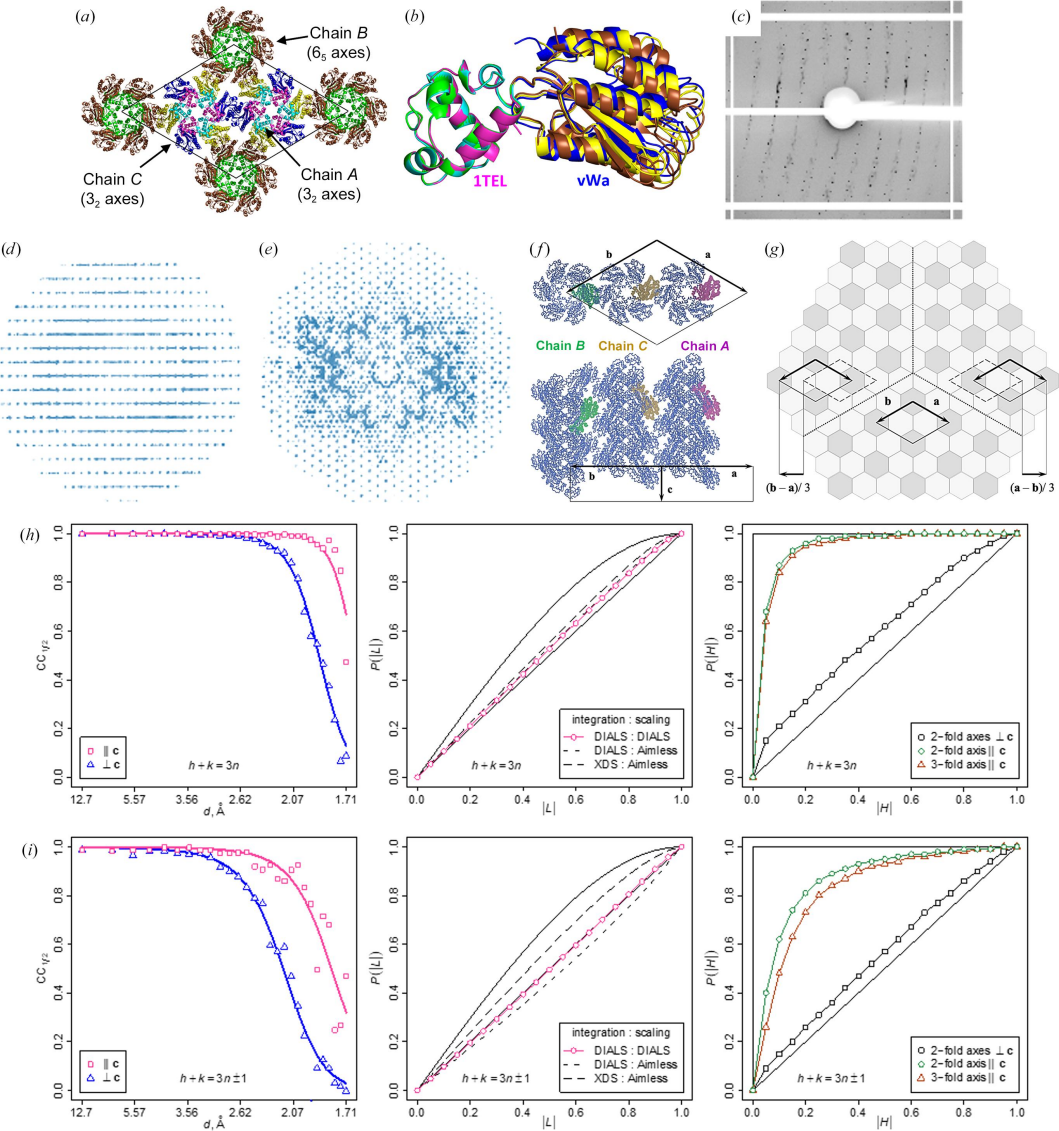
Possible models of crystal disorder. (*a*) Lattice contacts in the 1TEL-TV-vWa (SUMO) unit cell, with proteins shown in cartoon representation. Chain *A* (3_2_ axis) is coloured cyan (1TEL) and yellow (vWa). Chain *B* (6_5_ axis) is coloured green (1TEL) and brown (vWa). Chain *C* (3_2_ axis) is coloured magenta (1TEL) and dark blue (vWa). The unit cell is shown as a thin black line. (*b*) As in (*a*) but showing the chain *A*, *B* and *C* 1TEL-TV-vWa domains superimposed via their 1TEL domains. (*c*) Representative diffraction pattern of the 1TEL-TV-vWa (SUMO) crystal, showing diffuse scattering between the strong reflections. (*d*) Projection of reciprocal space viewed perpendicular to **c**. (*e*) As in (*d*) but viewed along **c**. (*f*) Packing ambiguity of adjacent helical polymers. (*g*) Possible model of the crystal architecture. The bright and dark hexagons represent 3_2_-axes (chains *A* and *C*) and 6_5_-axes (*B* chain) polymers, respectively. On the large scale, the difference between 3_2_-axes and 6_5_-axes polymers is that they are related by twofold rotation around an axis in the plane of the figure as shown in (*f*), and on a smaller scale the relative orientation of the 1TEL and vWa protein domains differs by 6.8° (chains *A* and *B*), 5.8° (chains *A* and *C*) and 11.7° (chains *C* and *B*). (*h*, *i*) Comparison of intensity statistics of reflection subsets. (*h*) Reflections *h* − *k* = 3*n*. (*i*) Reflections *h* − *k* = 3*n* ± 1. Left column: CC_1/2_ shows considerable differences in the useful resolution range of the two subsets. Centre column: the results of the *L*-test for twinning strongly depend on data processing. Right column: the results of *H*-tests are consistent between the two subsets of reflections and indicate no twinning relative to the twofold axis perpendicular to **c**. Thin solid black lines in the *L*-test plots represent theoretical curves for twinned (top) and nontwinned data (bottom). Thin black lines in the *H*-test plots represent the theoretical curve for the twinning operation or crystallographic symmetry operation (top line, step function) and the lattice symmetry operation unrelated to crystal symmetry or twinning (bottom line).

Some 1TEL–vWa crystals feature flipped TELSAM–target polymers. The 1TEL-TV-vWa (SUMO) crystal and data set selected for structure solution had a larger *P*6_5_ unit cell (*a* = *b* = 164.156, *c* = 54.409 Å) than other 1TEL-TV-vWa (SUMO) and 1TEL-AV-vWa (SUMO) crystals, similar to the unit-cell parameters of the batch 2 1TEL-AA-vWa (SUMO) crystal (*a* = *b* = 162.557, *c* = 56.877 Å; Fig. 1 ▸ *a*). This data set also exhibited significant translational noncrystallographic symmetry (tNCS; the largest off-origin peak in the Patterson map was 74.52% of the height of the origin peak). Molecular replacement with this data set was carried out as before, once again yielding a solution with 1TEL polymers along the 6_5_ axes and two additional 1TEL polymers along the two 3_2_ axes of the unit cell. In this case we did not see multiple orientations of the vWa domains relative to their host 1TEL polymers (Figs. 3 ▸ *a* and 3 ▸ *b*). For ease of explanation, we term these three chains chain *A* [1TEL polymers on the 3_2_ axes of the unit cell whose vWa domains interact with vWa domains from 1TEL polymers (chain *B*) on the 6_5_ axes], chain *B* [1TEL polymers on the 6_5_ axes of the unit cell whose vWa domains interact with vWa domains from 1TEL polymers (chain *A*) on the 3_2_ axes] and chain *C* (1TEL polymers on the 3_2_ axes whose vWa domains interact with their symmetry-equivalent chain *C* 3_2_-axis vWa domains). Our initial molecular-replacement solution featured parallel polymers that all ran in the same N→C direction, but we were not able to refine it to acceptable *R* factors (*R*_work_ and *R*_free_ values of 0.37 and 0.38, respectively). We next tried to re-index this data set into a smaller unit cell with 1TEL polymers only on the 6_5_ axes. This likewise gave only poor *R* factors (*R*_work_ and *R*_free_ values of 0.33 and 0.35, respectively). We attempted twin refinement in *phenix.refine* using the twin operator *h*, −*h* − *k*, −*l* (twofold axis parallel to **a**) for each unit-cell size, but this likewise yielded poor *R* factors.

We returned to the 1TEL-TV-vWa (SUMO) diffraction data set to identify the cause of our poor refinement statistics and of the signs of extreme tNCS (non-origin Patterson peak with a height 74.52% of the origin peak). The diffraction data for this 1TEL-TV-vWa (SUMO) crystal featured diffuse scattering between two out of every three reflections (Figs. 3 ▸ *c*–3 ▸ *e*). We hypothesized that this crystal is formed by small domains with *P*6_5_ symmetry packed with an offset of (*a* − *b*)/3 relative to their neighbours.

With this model, we attribute the diffuse patterns in the diffraction images (Figs. 3 ▸ *c*–3 ▸ *e*) to broken global periodicity in the crystal. In published structures with monoclinic ordered domains streaky diffraction spots were observed, with streaks extending in the direction of broken crystal periodicity and with rows of streaky reflections alternating with rows of well defined reflections in one of two other directions. Accordingly, the integrated intensities of reflections were modulated in the latter direction, with the period of modulation being non-commensurate with the reciprocal lattice. The diffraction images of the 1TEL-TV-vWa (SUMO) crystal show two-dimensional diffuse patterns in the planes perpendicular to c* (Fig. 3 ▸ *d*). The streaks connect weak reflections *h* − *k* = 3*n* ± 1, forming hexagonal shapes around well defined strong reflections *h* − *k* = 3*n*. Here, the modulation of intensities is consistent with a hexagonal lattice and the modulation coefficients take only two values: one applies to reflections *h* − *k* = 3*n* and the other to the reflections *h* − *k* = 3*n* ± 1. The high ratio of the two coefficients of about 7 (see Section 2.6) explains the high values of the *R* factors in refinements using all of the data without correction.

The structure and *P*6_5_ symmetry of the ordered domains were determined by molecular replacement using a subset of strong reflections and were later confirmed by molecular replacement against a complete but demodulated data set expanded to space group *P*1. The domains are formed by helical polymers of 1TEL-TV-vWa (SUMO) molecules, with the 6_5_-axes polymers coinciding with the crystallographic unit-cell axes. TELSAM helical polymers are present in two orientations: one is adopted by polymers lying on the 3_2_ crystallographic axes (further referred to as 3_2_-axes polymers) and the other by polymers lying on the 6_5_ axes (6_5_-axes polymers) (Fig. 3 ▸ *f*). This explains the high *R* factors from refinement in the small cell, wherein the model corresponds to a putative crystal packing with all polymer helices in the same orientation.

The 3_2_-axes polymers form a continuous interconnected framework, whereas the 6_5_ helices make no contact with each other. In the model in Fig. 3 ▸(*g*) these requirements are satisfied in each of the ordered domains and in the interfaces between them. Were it not for this constraint, there would be nothing to prevent some of the adjacent domains from twinning, However, the *H*-test for twofold axes perpendicular to **c** indicates the opposite (Figs. 3 ▸ *h* and 3 ▸ *i*, right panels). The fact that our initial twin refinement failed to improve the fit to the data also suggests that polymers with different N→C orientations are uniformly distributed over the crystal rather than aggregated into separate twin domains. High values of *R*_merge_ for these symmetry operations, above 0.55 for resolution cutoffs of 2.2 and 2.9 Å, are in agreement with the *H*-test. We thus favour the model in Fig. 3 ▸(*g*) with direct contacts between 6_5_-axes polymers prohibited.

The translation (*a* − *b*)/3 is a tNCS operation in the 1TEL-TV-vWa (SUMO) structure because it relates some 3_2_-axes polymers to some other 3_2_-axes polymers, but it is not a pseudosymmetry operation because it places 6_5_-axes polymers in the positions of 3_2_-axes polymers in an incorrect orientation. Therefore, the structure cannot be approximated by a structure with a three times smaller unit cell, and the correction of intensities was critical for structure determination. However, it is easy to imagine a structure with both pseudosymmetry (*a* − *b*)/3 and ordered domains offset by this operation. The structure solution in the small cell would then be possible and the electron-density maps would represent an average density from the three molecules related by pseudosymmetry. The weak reflections could still be observed, but refinement of a structure with correct unit-cell parameters to reasonable *R* factors would be impossible (without correction of intensities). It is likely that the difficulties with refinement in, for example, Ostrowski *et al.* (2015 ▸) and the unexplained weak reflections observed in, for example, Li *et al.* (2016 ▸) can be explained by crystal defects similar to those in the 1TEL-TV-vWa (SUMO) crystal. The corrected set of structure amplitudes has been deposited in the PDB along with the scaled unmerged data without correction. The ISa, *R*_work_ and *R*_free_ values for the 1/3 subset of strongest reflections *h* − *k* = 3*n* known to be least affected by partial crystal disorder are provided in Table 2 ▸ for reference.

We executed molecular replacement using both a version of the data set that included only the strongest 1/3 of the reflections and a version that included all reflections, with the strongest 1/3 of reflections having a different scale factor to the remaining 2/3 of reflections, both cut back to 1.9 Å resolution. Molecular replacement was executed as before for both versions of the data and produced essentially the same solution in each case, placing 1TEL polymers on both the 6_5_ and 3_2_ axes of the unit cell as before. As expected, the 1TEL polymers on the 6_5_ axes (chain *B*) of the unit cell ran in the opposite N→C direction from the polymers on the 3_2_ axes, consistent with our model of the overall crystal architecture (Figs. 3 ▸ *f* and 3 ▸ *g*). The vWa domains in this model were all fully resolved [in contrast to the batch 2 1TEL-AA-vWa (SUMO) structure described above] and all adopted the same binding mode to their host 1TEL polymers, regardless of the polymer orientation (Fig. 3 ▸ *b*). This binding mode was distinct from any of the vWa binding modes seen in either the batch 1 (PDB entry 7n1o) or batch 2 (PDB entry 8fzv) 1TEL-AA-vWa (SUMO) structures (Fig. 7*e*).

**Figure 4:**
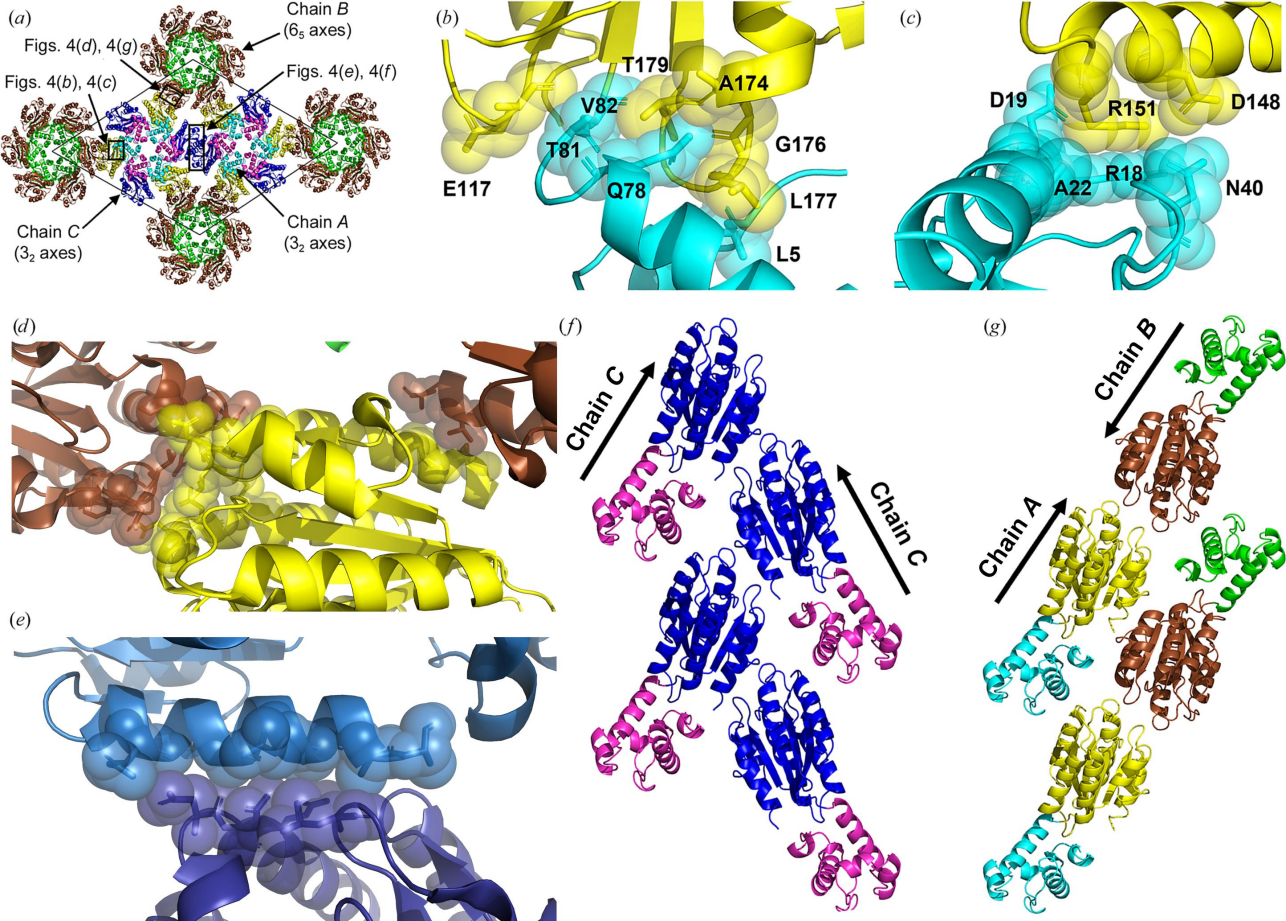
Molecular details of 1TEL-TV-vWa (SUMO). (*a*) Schematic of the unit cell, indicating the areas highlighted in subsequent panels. (*b*) Detail of the interaction between the chain *A* vWa domain (yellow) and its host 1TEL domain (cyan). The protein is shown in cartoon representation with selected side chains shown as sticks and transparent spheres. (*c*) As in (*b*) but for the interaction between the chain *A* vWa and a 1TEL domain from the next turn of the host polymer. (*d*) As in (*b*) but for the interaction between the chain *A* vWa domain (yellow) and two chain *B* vWa domains (brown). (*e*) As in (*b*) but for the interaction between the chain *C* vWa domain (light blue) and an adjacent, symmetry-related chain *C* vWa domain (dark blue). (*f*, *g*) Comparison of the antiparallel (*f*) polymer–polymer interaction between chain *A* (cyan and yellow) and chain *B* (green and brown) and the parallel (*g*) polymer–polymer interaction between two copies of chain *C* (magenta and dark blue).

We were able to refine the model against the 1/3 data set to an *R*_work_ and *R*_free_ of 0.23 and 0.28, respectively, and against the complete data set to an *R*_work_ and *R*_free_ of 0.24 and 0.27, respectively (Table 2 ▸). The vWa domains (using chain *A* as an example) adopt a binding mode to their host 1TEL domains that buries 326 Å^2^ of solvent-accessible surface area (average of both sides of the interface). Specifically, the side chains of Leu177, Gly176, Ala174 and Thr179 of the vWa domain interact with the side chains of Gln78, Thr81 and Val82 of the 1TEL domain (Figs. 4 ▸ *a* and 4 ▸ *b*). The chain *A* vWa domain makes glancing contacts with the chain *C* vWa domains on either side of it within its host polymer, as well as to the chain *A* 1TEL domain one turn of its host polymer above it. This last interface buries 188 Å^2^ of solvent-accessible surface area and is mostly polar and highly solvated. Specifically, this interface involves polar and van der Waals contacts between Asp148 and Arg151 of vWa and Arg18, Asp19, Ala22 and Asn40 of 1TEL (Fig. 4 ▸ *c*). The chain *A* vWa domain makes no contacts to the 1TEL domains of adjacent polymers, similar to the batch 1 1TEL-AA-vWa (SUMO) structure (PDB entry 7n1o), but in contrast to the batch 2 1TEL-AA-vWa (SUMO) structure.

1TEL-TV-vWa (SUMO) provides an example in which all target proteins adopt the same binding mode to their host polymers, but adjacent polymers adopt one of two binding modes to one another. This observation suggests that while the binding mode of vWa to its host 1TEL polymer can be selected at the time of polymer–polymer association, at least in the case of 1TEL-TV-vWa (SUMO) there exists a sufficiently low-energy binding mode such that all of the vWa domains reliably select the same binding mode, possibly even some time prior to polymer–polymer association.

To investigate the cause of TELSAM-polymer flipping in this crystal, we analysed the contacts made between parallel polymers and those made between antiparallel polymers. The 3_2_-axis chain *A* vWa domains make *C*2-symmetric ‘head-to-head’ interactions with 6_5_-axis chain *B* vWa domains, burying 341 Å^2^ of solvent-accessible surface area. This interface comprises a mixture of hydrogen bonds, hydrophobic interactions and van der Waals interactions. Specifically, Ser94, Asn97, Asn98, Glu101, Leu219, Asp220 and Phe221 in the chain *A* vWa domain contact the very same set of residues in the chain *B* vWa domain. Numerous well ordered water molecules are also seen both within and around the periphery of this interface (Fig. 4 ▸ *d*).

Along the length of the TELSAM polymers, the 3_2_-axis chain *C * vWa domains adopt a zipper-like arrangement, with each vWa domain contacting two other chain *C* vWa domains from an adjacent 3_2_-axis polymer. Each vWa domain–vWa domain interaction buries 209 Å^2^ of solvent-accessible surface area and consists mostly of hydrophobic and van der Waals contacts between the side chains and main chains of Gln244, Gly248, Asn251, Ser252 and Ala255 of one vWa domain and of Asn97, Asn98 and Leu219 of the second vWa domain. Numerous well ordered water molecules and a sulfate ion are also seen around the periphery of this interface (Fig. 4 ▸ *e*). When the solvent content of the antiparallel vWa chain *A* to vWa chain *B* interface is considered, we propose that this interface has a similar binding energy to the more hydrophobic parallel chain *C* to chain *C* interface, enabling the observed polymer-flipping phenomenon (Figs. 3 ▸ *f*, 4 ▸ *f* and 4 ▸ *g*). Similar pathologies were also seen in other 1TEL-TV-vWa (SUMO) and 1TEL-AV-vWa (SUMO) crystals (Table 1 ▸).

### 3.3. Replacing the cleavable 10×His-SUMO tag with a noncleavable 10×His tag significantly improved the diffraction resolution and correlates with an absence of TELSAM-polymer flipping

Decreasing the flexibility of the 1TEL–vWa linker moderately improved the diffraction resolution but failed to prevent other pathologies, such as twinning, tNCS and TELSAM-polymer flipping. We have observed that trace proteases can cleave target proteins from the TELSAM polymer and that this can have profound effects on TELSAM polymerization and crystallization dynamics and architecture. We have also observed that omitting the cleavable SUMO tag and appending the 10×His tag directly to the N-terminus of TELSAM can change the crystal form and diffraction characteristics (unpublished data).

To investigate the effects of omitting the SUMO protease during purification and leaving the 10×His tag appended to the N-terminus of 1TEL, we remade two genes, for 1TEL-AA-vWa and 1TEL-TV-vWa, without the SUMO tag. We fused the 10×His tag directly to the N-terminus of 1TEL and retained the tag through purification and into the crystallization trays. We expressed, purified and crystallized both proteins at 1 and 20 mg ml^−1^ with different commercially available and custom-made crystallization screens. His-1TEL-TV-vWa formed crystals in 2–4 days, while His-1TEL-AA-vWa took seven days to crystallize. Some His-1TEL-AA-vWa crystals were soaked with iodine before harvesting (Miyatake *et al.*, 2006 ▸), while the His-1TEL-TV-vWa crystals were harvested without iodine treatment. Omission of the SUMO tag correlated with improved diffraction resolution of the His-1TEL-AA-vWa and His-1TEL-TV-vWa crystals by 0.4 and 0.7 Å, respectively. No twinning, pseudo-translation or TELSAM-polymer flipping was detected in diffraction data sets from these SUMO tag-free constructs.

**Figure 5:**
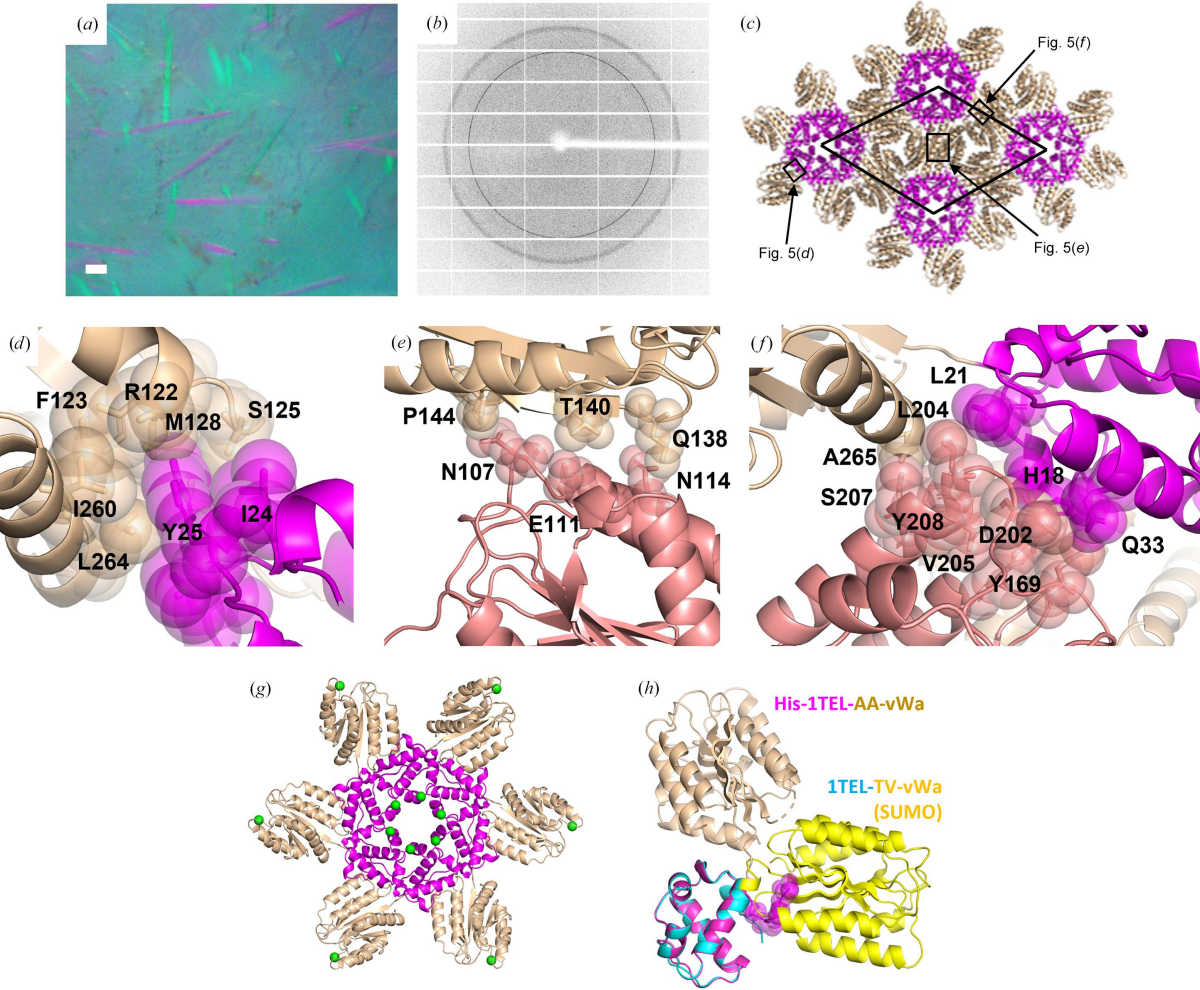
Crystallization and structural details of His-1TEL-AA-vWa. (*a*) Representative crystals of His-1TEL-AA-vWa. The scale bar is 100 µm. (*b*) Representative diffraction pattern from a His-1TEL-AA-vWa crystal. (*c*) Unit-cell packing of a His-1TEL-AA-vWa crystal with the 1TEL domain in magenta and the vWa domain in wheat. The unit cell is indicated by a thin black line. (*d*) Detail of the interaction between a vWa domain (wheat) and its host 1TEL domain (magenta). The protein is shown in cartoon representation with selected side chains shown as sticks and transparent spheres. (*e*) As in (*d*) but showing the interaction between the vWa domain (salmon) and a vWa domain (wheat) from an adjacent TELSAM polymer. (*f*) As in (*d*) and (*e*) but highlighting the contacts made by a vWa domain to a second vWa domain and its host 1TEL domain. (*g*) As in (*d*) but highlighting the position of the iodinated tyrosine residues (small green spheres) relative to the 1TEL–vWa polymer. (*h*) As in (*d*) but highlighting (magenta spheres) the N-terminal 10×His tag of the 1TEL domain. The structure of chain *A* of the 1TEL-TV-vWA (SUMO) structure is superimposed via its 1TEL domain and shown in cyan (1TEL) and yellow (vWa).

His-1TEL-AA-vWa formed around 50 crystals averaging 300 µm in length (Fig. 5 ▸ *a*). The average resolution of these His-1TEL-AA-vWa crystals was 2.6 Å (with a range of 2.5–2.8 Å across four crystals), which was somewhat better than that of 1TEL-AA-vWa (SUMO), which was 2.9 Å (with a range of 2.8–3.0 Å across three crystals) (Fig. 5 ▸ *b*, Table 1 ▸). Crystals of His-1TEL-AA-vWa indexed an average of 67% of non-ice reflections (range 54–94%) and exhibited an average mosaicity of 0.16° (range 0.07–0.21°) and an average ISa of 19 (range 15–23). Molecular replacement yielded a solution with 1TEL polymers along the sixfold axes of the *P*6_5_ unit cell, with unit-cell parameters *a* = *b* = 99.7, *c* = 50.1 Å (Fig. 5 ▸ *d*). The vWa domains of His-1TEL-AA-vWa adopt a binding mode against their host 1TEL domains that buries 269 Å^2^ of solvent-accessible surface area. Specifically, the side chains of Arg122, Phe123, Ser125, Met128, Ile260 and Leu264 of the vWa domain interact with the side chains of Tyr25 and Ile24 of the 1TEL domain (Fig. 5 ▸ *d*). The interface of vWa with the vWa domain of an adjacent polymer buries 188 Å^2^ of solvent-accessible surface area. Specifically, the side chain of Gln138 in the vWa domain interacts with the side chain of Asn114 in the adjacent vWa domain. Additionally, the side chains of Glu111 and Asn107 in the vWa domain interact with the side chains of Pro144 and Thr140 in the adjacent vWa domain (Fig. 5 ▸ *e*). The vWa domain also contacts the 1TEL domain of an adjacent polymer, burying 270 Å^2^ of solvent-accessible surface area. Specifically, the side chains and main chains of Tyr208, Val205, Asp202, Leu204 and Tyr169 in the vWa domain contact the side chains and main chains of Leu21, His18 and Gln33 in the 1TEL domain (Fig. 5 ▸ *f*). The vWa domain also contacts a second vWa domain, burying 144 Å^2^ of solvent-accessible surface area. Specifically, the side chain of Ser207 in the vWa domain makes weak contacts with the side chain of Ala265 in this second vWa domain (Fig. 5 ▸ *f*).

Prior to cooling, this crystal was briefly (∼10 s) exposed to iodine vapour (Miyatake *et al.*, 2006 ▸) to label exposed tyrosine side chains. Our object was to assess the degree and location of potential iodine labelling sites on 1TEL for use with target proteins for which molecular replacement is unsuccessful. We observed iodine labelling with 79% occupancy of only the less solvent-exposed C^ɛ^ atom of Tyr69 of the 1TEL domain, which faces into the solvent void at the centre of the 1TEL polymer (Fig. 5 ▸ *g*). We also observed 64% iodine labelling of only one of the C^ɛ^ atoms of Tyr208 of the vWa domain, which makes direct contact with the 1TEL domain of an adjacent 1TEL polymer (Fig. 5 ▸ *g*).

We were also able to resolve the main-chain atoms of two of the histidine residues in the 10×His tag. These histidine residues pack loosely against the main chains of Arg149 and Ser153 of the vWa domain from an adjacent 1TEL polymer. Together with the fact that inclusion of the 10×His tag did not appear to negatively impact crystal nucleation or growth, this suggests that the His tag did not interfere with 1TEL polymerization or target proteins docking to their host polymers. Notably, the vWa domains adopt a binding mode to their 1TEL polymers that is distinct from that observed for any previous 1TEL–vWa construct, possibly due to the His tag favouring this binding mode or disfavouring the previously observed binding modes. To test this, we superimposed our previous 1TEL-TV-vWA (SUMO) chain *A* structure with this His-1TEL-AA-vWa structure through their 1TEL domains. This superposition revealed that the His tag occupies the same space as that occupied by the vWa domain of the previous 1TEL-TV-vWa (SUMO) structure, thus potentially forcing the vWa domain of 1TEL-AA-vWa into an alternative position relative to its host 1TEL domain (Fig. 5 ▸ *h*). In the light of our prior hypothesis that the vWa binding mode of the 1TEL-TV-vWa (SUMO) construct may be the cause of the polymer-flipping pathology, this provides a possible mechanism by which omission of the cleavable SUMO tag from the 1TEL–vWa constructs prevents these pathologies: the presence of the noncleavable 10×His tag blocks access to the vWa binding mode which allows equally energetic parallel and antiparallel polymer–polymer association (and thus polymer flipping).

### 3.4. A bulkier TELSAM–target linker did not alter the crystal-packing interactions relative to a more flexible linker but did improve the diffraction resolution of 1TEL–vWa crystals

**Figure 6:**
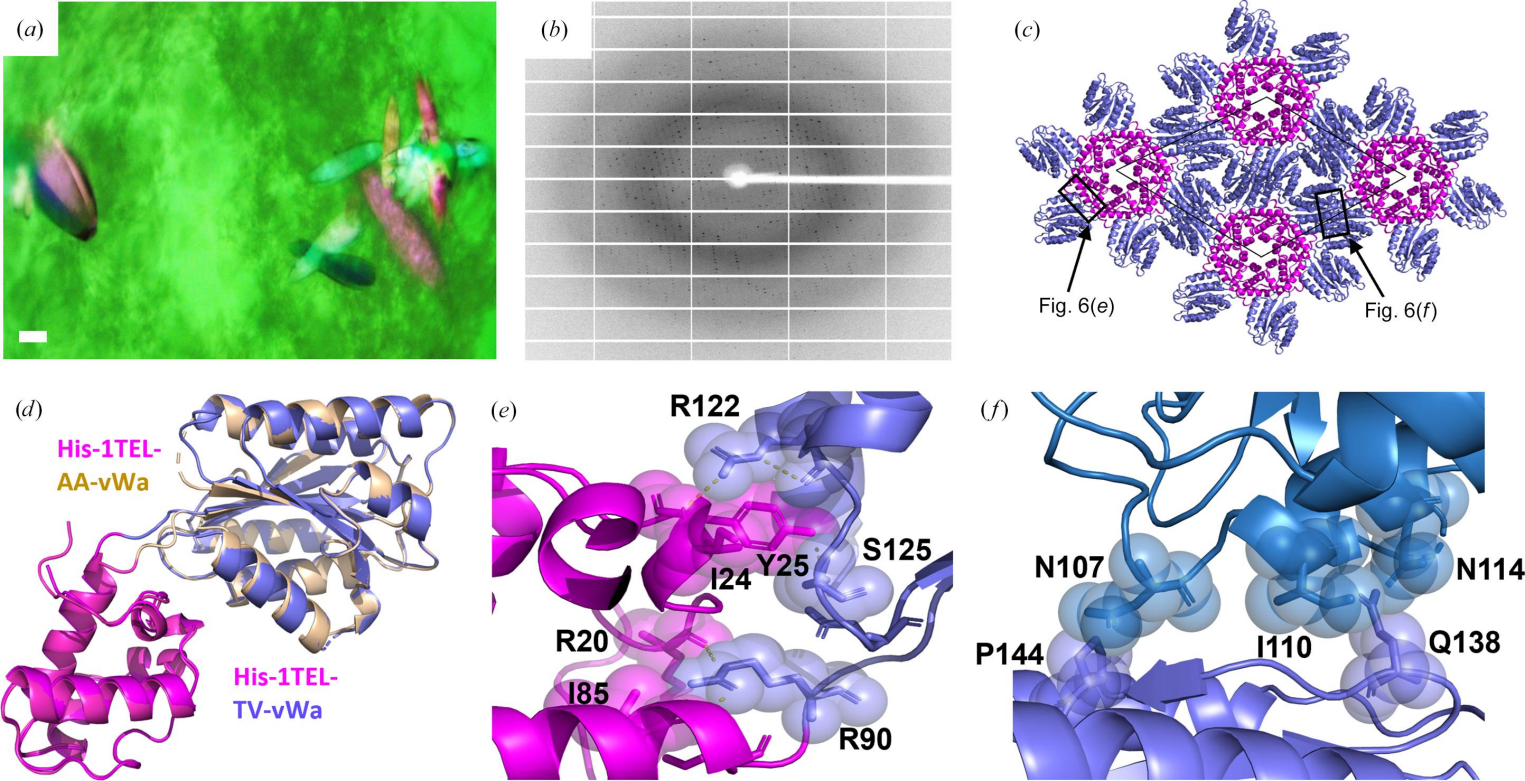
Crystallization and structural details of His-1TEL-TV-vWa. (*a*) Representative crystals of 1TEL-TV-vWa. The scale bar is 100 µm. (*b*) Representative diffraction pattern from a His-1TEL-TV-vWa crystal. (*c*) Unit-cell packing of a His-1TEL-TV-vWa crystal with the 1TEL domain in magenta and the vWa domain in slate. The unit cell is shown as a thin black line. (*d*) Superposition of the structure of His-1TEL-AA-vWa (magenta and wheat) with the structure of His-1TEL-TV-vWa (magenta and slate) on their 1TEL domains. (*e*) Detail of the interaction between the vWa domain (slate) and its host 1TEL domain (magenta). The protein is shown in cartoon representation with selected side chains shown as sticks and transparent spheres. (*f*) As in (*e*) but showing the interaction between the vWa domain (slate) and a vWa domain (blue) from an adjacent polymer.

His-1TEL-TV-vWa formed around 400 crystals (Fig. 6 ▸ *a*), which is equivalent to the number of crystals formed by 1TEL-AV-vWa (SUMO) and 1TEL-TV-vWa (SUMO) and greater than that formed by 1TEL-AA-vWa (SUMO) (PDB entry 7n1o) and His-1TEL-AA-vWa. His-1TEL-TV-vWa formed crystals that diffracted to an average resolution of 2.0 Å (with a range of 1.6–2.6 Å across 16 crystals), significantly higher than that of 1TEL-TV-vWa (SUMO), which was 2.7 Å (with a range of 2.3–3.0 Å), and the highest of all 1TEL–vWa fusions tested in this study. The crystals of His-1TEL-TV-vWa indexed an average of 77% of non-ice reflections (range 31–97%), exhibited an average mosaicity of 0.25° (range 0.12–0.40°) and had an average ISa of 25 (range 11–42) (Fig. 6 ▸ *b*, Table 1 ▸). In a subsequent crystallization concentration study, crystals of His-1TEL-TV-vWa were observed at concentrations as low as 2 mg ml^−1^ and at pH values between 5.0 and 8.5. Notably, crystals from this second batch of 1TEL-TV-vWa produced by the same research team also exhibited no signs of tNCS, twinning or TELSAM-polymer flipping.

Molecular replacement of His-1TEL-TV-vWa was carried out as above, yielding a solution with 1TEL polymers along the sixfold axes of the *P*6_5_ unit cell, with *a* = *b* = 100.9, *c* = 49.8 Å (Fig. 6 ▸ *c*). The binding modes of vWa to the host 1TEL polymers and the overall crystal packing were very similar to those observed for the His-1TEL-AA-vWa construct (Fig. 6 ▸ *d*). For example, the vWa domains of His-1TEL-TV-vWa adopt a binding mode to their host 1TEL domains that buries 470 Å^2^ of solvent-accessible surface area. Specifically, the side chains of Arg122 and Ser125 of the vWa domain make polar interactions with the side chain of Tyr25 of the 1TEL domain. Arg90 in the vWa domain also makes polar interactions with Arg20 and Ile85 in the 1TEL domain. Unlike His-1TEL-AA-vWa, His-1TEL-TV-vWa additionally makes many polar interactions between the vWa domain and its own 1TEL domain, which presumably make the orientation of the vWa domain more stable, potentially leading to an improved diffraction resolution (Fig. 6 ▸ *e*). The vWa domain makes glancing contacts with a second vWa domain, burying 240 Å^2^ of solvent-accessible surface area. Specifically, the side chain of Gln138 in the vWa domain forms van der Waals contact with the side chains of Ile110 and Asn114 in the adjacent vWa domain. Pro144 in the vWa domain also interacts weakly with Asn107 in the adjacent vWa domain (Fig. 6 ▸ *f*).

**Figure 7:**
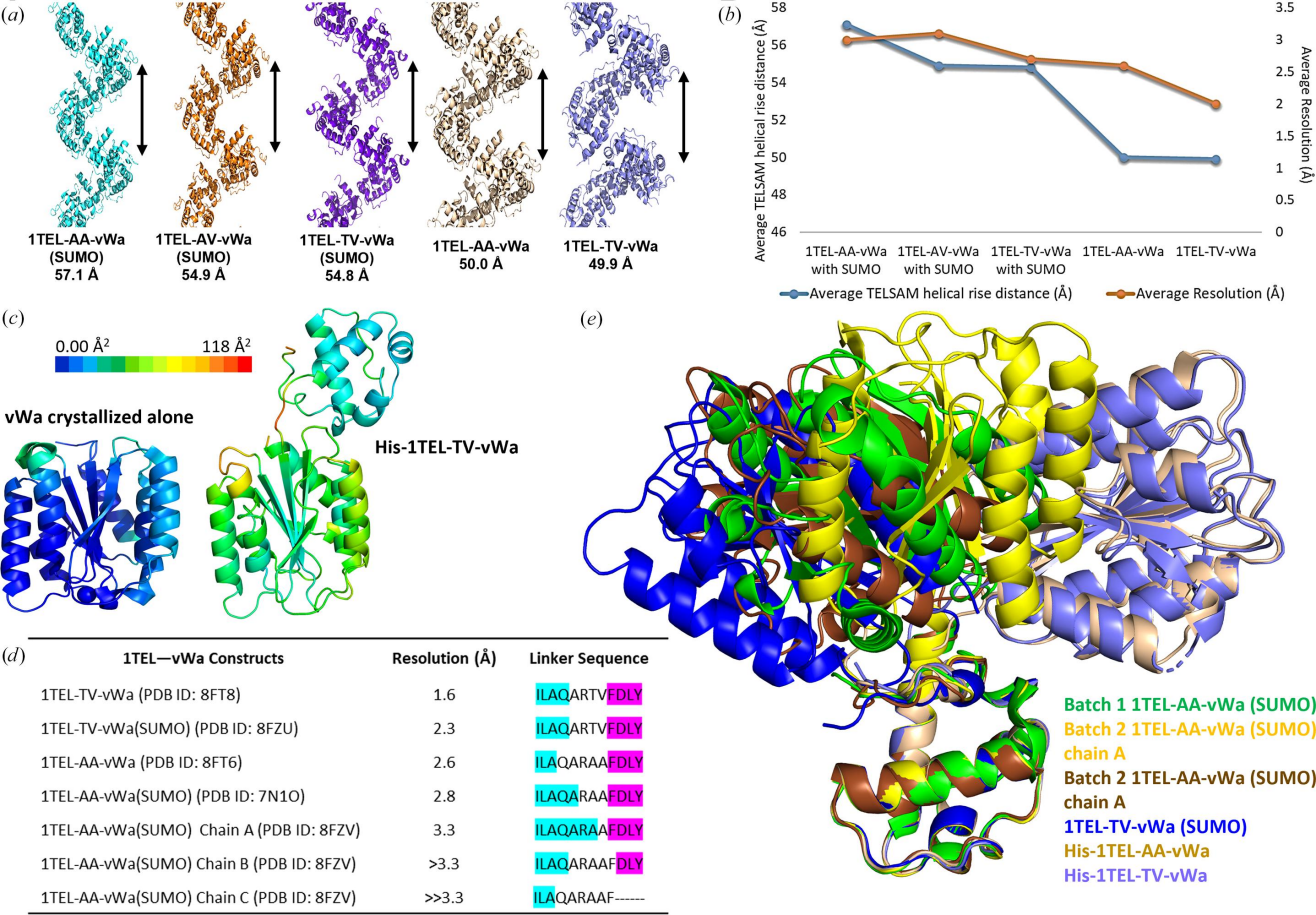
Comparison of the structures analysed in this study. (*a*) Schematic of the average helical rise of the 1TEL–vWa constructs discussed in this study. The relative rise of a single turn of each helix is denoted by a black arrow. Fused vWa domains are not shown. (*b*) Plot of the average resolution (orange line) and helical rise (blue line) of the constructs described in this study. (*c*) The highest resolution structure of the CMG2 vWa domain, PDB entry 1shu (1.5 Å resolution, left), and 1TEL-TV-vWa (1.6 Å resolution, right) are shown in cartoon representation and coloured according to the crystallographic *B* factor. The *B*-factor colour scale is given above the images and is the same in the two images. (*d*) Linker sequences for the constructs used in this study. Residues in the 1TEL C-terminal α-helix are highlighted in cyan, residues in the vWa N-terminal β-sheet are highlighted in magenta and residues that are in neither of these secondary structures are uncoloured. (*e*) Superposition of batch 1 1TEL-AA-vWa (SUMO) (green), batch 2 1TEL-AA-vWa (SUMO) chain *A* (yellow) and chain *B* (brown), 1TEL-TV-vWA (SUMO) (blue), His-1TEL-AA-vWa (wheat) and His-1TEL-TV-vWa (slate) on their 1TEL domains.

We compared the TELSAM helical rise in the 1TEL-AA-vWa (SUMO), 1TEL-AV-vWa (SUMO), 1TEL-TV-vWa (SUMO), His-1TEL-AA-vWa and His-1TEL-TV-vWa crystals to identify relationships between helical rise, linker composition, SUMO inclusion and diffraction resolution. We observed that the helical rise decreased with an increasing bulk of the amino acids forming the 1TEL–vWa connection (postulated to correlate with the rigidity of the linker) in the cases of 1TEL-AA-vWa (SUMO), 1TEL-AV-vWa (SUMO) and 1TEL-TV-vWa (SUMO). The same trend was also seen in the cases of His-1TEL-AA-vWa and His-1TEL-TV-vWa. We observed that constructs utilizing a SUMO tag had higher helical rises than constructs without a SUMO-tag fusion (Fig. 7 ▸ *a*). The decrease in helical rise also correlates with an improvement in diffraction resolution, which is to be expected. Increased unit-cell compactness and lower solvent content would be expected to correlate with increased intermolecular contacts and better resolution (Figs. 7 ▸ *a* and 7 ▸ *b*; Table 1 ▸).

1TEL-TV-vWa has a comparable diffraction resolution (1.6 Å) to that of the previous highest resolution structure of the vWa domain, PDB entry 1shu (1.5 Å). This is particularly notable because in our hands crystals of vWa alone diffracted to an average resolution of 2.4 Å (with a range of 1.9–3.1 Å across 18 crystals), index an average of 56% of non-ice reflections (range 19–95%) and exhibit an average mosaicity of 0.30° (range 0.20–0.45°) and an average ISa of 38 (range 23–53). For comparison, we solved and refined the structure of one of these vWa alone constructs at 2.19 Å resolution (PDB entry 8fz4). Data-processing and refinement statistics for this structure are given in Table 2 ▸. This observation suggests that fusion to 1TEL improved the resolution of vWa crystals produced in our hands by an average of 0.4 Å, a significant improvement (Table 1 ▸), providing an exciting hint into what more experienced crystallographers might be able to accomplish with TELSAM-mediated crystallization, such as in a recent stunning example involving the SARS-CoV2 nsp14 N7-methyltransferase domain (Kottur *et al.*, 2022 ▸).

Having a 1.6 Å resolution 1TEL–vWa fusion structure allowed us to directly compare the refined *B* factors between this structure and the previous best-resolution structure. PDB entry 1shu (Lacy *et al.*, 2004 ▸) had an average *B* factor of 21.1 Å^2^ (with a range of 0.0–65.9 Å^2^) across just the protein atoms. The vWa domain in the 1TEL-TV-vWa construct had an average *B* factor of 46.6 Å^2^ (with a range of 22.8–97.2 Å^2^), while the 1TEL domain had an average *B* factor of 35.1 Å^2^ (with a range of 18.4–96.3 Å^2^) and the linker between them had an average *B* factor of 60.4 Å^2^ (with a range of 35.3–99.6 Å^2^) (Fig. 7 ▸ *c*). This result confirms our earlier hypothesis (Nawarathnage *et al.*, 2022 ▸) that fusion to 1TEL allows the formation of crystals with a comparable diffraction resolution but increased molecular motion in the crystal lattice. Concomitant with this, the solvent content of the highest-resolution crystal of the vWa domain crystallized on its own was 47.22% (PDB entry 1shu), while the solvent content of His-1TEL-TV-vWa crystals was 49.46% (PDB entry 8ft8). Of note, the crystals of the vWa domain crystallized on its own in our hands had a solvent content of 40.76% (PDB entry 8fz4), while the batch 1 1TEL-AA-vWa (SUMO) crystals had a solvent content of 59.59% (PDB entry 7n1o). We anticipate that the increased solvent content of TELSAM-fusion crystals may facilitate drug-soaking experiments. We demonstrated this as described above by soaking diatomic iodine into crystals of His-1TEL-AA-vWa. When soaking 1TEL-AA-vWa (SUMO) and later His-1TEL-TV-vWa crystals with the drug candidate PGM, we observed that PGM becomes yellow upon air exposure and that 1TEL–vWa crystals also became yellow after an overnight soak with PGM, suggesting that the molecule had entered the solvent channels of the crystal. In contrast, crystals of the vWa domain crystallized on its own did not take up the yellow colour after an overnight soak with PGM, suggesting that these crystals do not take up the PGM molecule as readily as the 1TEL–vWA crystals.

We sought a structural explanation of how increasing the side-chain bulk of linker amino acids might improve the resolution of 1TEL–vWa constructs. Increasing the side-chain bulk of linker amino acids from Ala-Ala to Thr-Val correlated with a modest improvement in the resolution of 1TEL–vWa (SUMO) and His-1TEL–vWa constructs by 0.3 and 0.6 Å, respectively. Our structures reveal that substitutions of the amino acids in the linker do not make it fully rigid, but rather appear to reduce the number of highly flexible residues that are present. We note that in all 1TEL–vWa structures solved to date there is always at least one amino acid in the linker that is not part of the 1TEL C-terminal α-helix or the vWa N-terminal β-sheet, such as in batch 1 (PDB entry 7n1o; Nawarathnage *et al.*, 2022 ▸) or batch 2 1TEL-AA-vWa (SUMO) chain *A* (Fig. 7 ▸ *d*). This observation may suggest that a fully rigid connection between 1TEL and a target protein is not favourable for crystal formation, as we had suggested previously. This is a property that we are testing in more depth in a follow-up study.

Alignment of the 1TEL domains of all 1TEL–vWa structures solved to date reveals that there is some correlation between the number of linker residues that are not part of the 1TEL C-terminal α-helix or the vWa N-terminal β-sheet and the observed or extrapolated (from the resolution and lack of crystal defects) order of the fused vWa domain (Fig. 7 ▸ *d*). For example, His-1TEL-TV-vWa had four such amino acids and yielded a 1.6 Å resolution structure, while His-1TEL-AA-vWa had five such amino acids and yielded a 2.6 Å resolution structure. Likewise, in the batch 2 1TEL-AA-vWa (SUMO) structure chain *A* had one linker amino acid that was not in a regular secondary structure and had a resolution of <3.3 Å, while chain *B* had five such linker amino acids and a resolution of ∼3.3 Å and chain *C* had six such linker amino acids and a resolution of ≫3.3 Å. We further note that in structures of 1TEL-TV-vWa constructs with or without SUMO, the valine side chain of the Thr-Val linker segment is nearly always well resolved in the electron density, while the threonine never is, suggesting that it adopts multiple side-chain conformational states. This fits well with the 1TEL-AV-vWa (SUMO) linker variant crystallizing nearly as well as the 1TEL-TV-vWa (SUMO) variant, while the 1TEL-TT-vWa (SUMO) variant did not form crystals.

The 1TEL–vWa constructs characterized to date reveal that the vWa domains appear to be able to adopt at least nine distinct binding modes to their host polymers, four of which were clearly observed and five of which were extrapolated from partial electron density (Fig. 7 ▸ *e*). Interestingly, the choice of vWa binding mode showed no correlation with the type of linker used (Fig. 7 ▸ *d*), but was strongly correlated with the presence or absence of the SUMO tag and/or the batch of protein. Curiously, 1TEL-AV-vWa (SUMO) (to be described elsewhere) and 1TEL-TV-vWa (SUMO) were prepared in separate batches by the same research team but exhibited the same vWa binding mode. Likewise, His-1TEL-AA-vWa and His-1TEL-TV-vWa were prepared in separate batches by the same research team but also exhibited the same vWa binding mode, which was distinct from that of 1TEL-AV-vWa (SUMO) and 1TEL-TV-vWa (SUMO). Taken together, these observations highlight the still very significant factor of batch-to-batch variation and researcher skill level on crystallization dynamics and thus on crystallization propensity and quality, regardless of whether TELSAM fusion is employed.

## 4. Discussion

Fusion to TELSAM has now been demonstrated in multiple cases to allow the rapid crystallization of even difficult target proteins (Kottur *et al.*, 2022 ▸), to enable high-resolution data collection (<2.0 Å), to allow crystallization at unusually low protein concentrations and to result in crystals where the protein of interest has an increased degree of residual molecular motion, as shown by significantly higher crystallographic *B* factors. These higher *B* factors hint that TELSAM-mediated crystals may capture proteins of interest in subtly more physiological conformations, with less interference from crystal-packing artefacts. If this is true, then it may open the door to higher accuracy studies of protein structure and dynamics, potentially benefitting fields such as protein and enzyme engineering and the study of intrinsically disordered and partially folded proteins. Due to built-in validations of TELSAM (TELSAM subunits most often form the expected sixfold helical polymer and the target proteins must be within an appropriate distance of the C-terminus of TELSAM), TELSAM-fusion crystals are a promising system to enable phase solution and structure determination of such difficult proteins.

In replicating our previous results, we identified and investigated several parameters that were unexplored in our initial pilot study (Nawarathnage *et al.*, 2022 ▸), as well as previously unencountered issues, such as target proteins that are not fully resolved in the resulting electron density. To better understand the best practices for TELSAM-mediated crystallization and improve the reliability of the technique, we experimented with modifications to the 1TEL–vWa linker and found that doing so modestly improved the diffraction resolution and appeared to enforce a single mode of vWa-domain binding to the host 1TEL polymer. Based on current evidence, we propose that the optimal linker between 1TEL and a protein of interest may consist of a small number (∼2) of minimally flexible residues that place the protein of interest as close to the 1TEL polymer as possible.

The fact that three of the four linkers tested in this study resulted in crystallization and that some linkers clearly outperformed others suggests the utility of designing and testing a few different linker variants to optimize the chances of crystallization and of obtaining a high-resolution (<2.0 Å) structure of a protein of interest. This parallels the experience of Kottur and coworkers, where three different linkers were tested in an attempt to crystallize the recalcitrant SARS CoV2 nsp14 N7-methyltransferase. In this case, only one of the three linkers resulted in crystals, but with optimization of the crystallization conditions the authors were able to reliably achieve a diffraction resolution of 1.4–1.6 Å (Kottur *et al.*, 2022 ▸).

We have identified the phenomenon of TELSAM-polymer flipping and were able to largely correct for it by differently scaling the weak and strong reflections in our data. The approach used to correct the data could provide a way forward for other systems that exhibit similar diffraction data pathologies. We observed that polymer flipping appears to correlate with the specific binding mode of the target protein to its host TELSAM polymer. Based on this observation, we hypothesize that polymer flipping is possible whenever the fused protein of interest adopts a binding mode to its host TELSAM polymer that allows parallel and antiparallel TELSAM polymer–polymer interactions with a roughly similar binding energy. While the inclusion of a noncleavable 10×His tag appears to have blocked the vWa-domain binding mode associated with TELSAM-polymer flipping in the current case, we expect that polymer flipping may be dependent on the specific size, shape and preferred binding mode of the target protein. This phenomenon and its effective treatment are the subjects of ongoing study.

Based on the minimal contacts observed between the vWa domain and its host or adjacent 1TEL polymers, we hypothesize that target proteins choose their binding mode to their host TELSAM polymers at the time of polymer–polymer association. We also hypothesize that to obtain a high-resolution structure with a well resolved target protein, all target proteins must adopt the same binding modes to their host polymers throughout the crystal and TELSAM–target polymers must then adopt a single binding mode to one another. The somewhat lower fraction of reflections indexed in some TELSAM-fusion crystals may hint at a failure to completely achieve these two requirements throughout the entirety of these crystals, although this has not prevented high-resolution structure solution in the cases reported here.

We provide evidence that retention of the 10×His tag significantly altered the binding mode of the target protein to its host 1TEL polymer, the crystal lattice interactions and the degree of order in the resulting crystal. In the constructs reported here, retaining the 10×His tag during crystallization clearly did not block crystal formation and may have improved the crystal order by limiting the possible binding modes of the target protein to its host TELSAM polymer. Based on these observations, we currently recommend considering both the retention of His tags and their proteolytic removal when designing TELSAM–target protein fusion constructs. We are currently testing this important variable with constructs involving other target proteins.

As to the universality of the method, thus far we have observed that in our hands TELSAM fusion crystallized seven out of eight proteins of interest, but that three of these seven have not yet resulted in a solved structure. We have evidence that the polymer-flipping phenomenon may be responsible for at least some of these cases. Recent results by Kottur *et al.* (2022 ▸) reveal that not all TELSAM–target protein linkers are equally viable, a result that is confirmed by the current study. We expect that as we refine the requirements for TELSAM-fusion crystallization (for example linkers) and develop more straightforward methods to correct data sets arising from flipped polymer crystals, the solved structure success rate of TELSAM-fusion crystallography will increase.

## Supplementary Material

PDB reference: 1TEL-AA-vWa, 8ft6

PDB reference: 1TEL-TV-vWa, 8ft8

PDB reference: 1TEL-TV-vWa (SUMO), 8fzu

PDB reference: 1TEL-AA-vWa (SUMO), 8fzv

PDB reference: vWa alone, 8fz4

## Figures and Tables

**Table 1:** Crystallization time, propensity and diffraction quality of vWa constructs

Construct	Days to crystal appearance	No. of commercial conditions giving crystals	No. of commercial conditions screened	Diffraction limit of collected data sets (Å)	Fraction of non-ice reflections indexed (%)	Average maximum mosaicity used in data processing (°)	Final ISa of data processing	Unit-cell solvent content (%)	Largest off-origin Patterson peak (%)	Twin fraction (%)
1TEL-AA-vWa (SUMO) (batch 1)†	3–10	9 (3%)	288	2.9, *n* = 3 (2.8–3.0)	57 (43–67)	0.47 (0.38–0.60)	16 (15–17)	59.6	5.06	0.00
VWa alone†	35–39	4 (1%)	384	2.4, *n* = 18 (1.9–2.9)	56 (19–95)	0.30 (0.20–0.45)	38 (23–53)	40.8	9.18	0.00
1TEL-AV-vWa (SUMO)	3–10	4 (0.8%)	480	2.9, *n* = 8 (2.5–3.5)	35 (12–73)	0.59 (0.23–1.10)	20 (6–36)	ND	6.26	0.425
1TEL-TV-vWa (SUMO)	3–10	4 (0.8%)	480	2.7, *n* = 6 (2.3–3.0)	49 (15–88)	0.55 (0.18–1.10)	19 (7–34)	49.9	74.52	0.047
His-1TEL-AA-vWa	7–10	2 (0.4%)	480	2.6, *n* = 4 (2.5–2.8)	67 (54–94)	0.16 (0.07–0.21)	19 (15–23)	50.5	4.37	0.027
His-1TEL-TV-vWa	2–7	4 (0.8%)	480	2.0, *n* = 16 (1.6–2.6)	77 (31–97)	0.25 (0.12–0.40)	25 (11–42)	49.5	4.65	0.005

† Data taken from Nawarathnage *et al.* (2022 ▸).

**Table 2:** Data-collection and refinement statistics Values in parentheses are for the highest resolution shell.

Construct	Batch 2 1TEL-AA-vWa (SUMO)	1TEL-TV-vWa (SUMO)	1TEL-TV-vWa (SUMO) (1/3 of data)	His-1TEL-AA-vWa	His-1TEL-TV-vWa	vWa alone
PDB code	8fzv	8fzu		8ft6	8ft8	8fz4
X-ray source	SSRL BL12-1	SSRL BL12-2	SSRL BL12-2	SSRL BL12-2	SSRL BL12-2	SSRL BL9-2
Wavelength (Å)	0.979460	1.00000	1.00000	1.85321	0.979460	0.979460
Temperature (K)	100	100	100	100	100	100
Detector distance (mm)	400	300	300	250	300	250
Resolution range (Å)	70.39–3.292 (3.409–3.292)	71.06–1.90 (1.968–1.900)	82.04–1.90	43.19–2.62 (2.714–2.620)	19.87–1.60 (1.658–1.600)	39.08–2.19 (2.268–2.190)
Space group	*P*6_5_	*P*6_5_	*P*6_5_	*P*6_5_	*P*6_5_	*C*121
*a*, *b*, *c* (Å)	162.557, 162.557, 56.877	164.096, 164.096, 54.4009	164.096, 164.096, 54.4009	99.745, 99.745, 50.135	100.882, 100.882, 49.754	78.244, 88.969, 59.779
α, β, γ (°)	90, 90, 120	90, 90, 120	90, 90, 120	90, 90, 120	90, 90, 120	90, 128.127, 90
Total reflections	101038 (10665)	668275 (67759)	308068 (15372)	296744 (28503)	724353 (74091)	113715 (11309)
Unique reflections	12820 (515)	66252 (2186)	30824 (1592)	8712 (861)	38098 (3766)	16403 (1620)
Multiplicity	7.9 (8.1)	10.1 (10.3)	10.0 (9.7)	34.1 (33.0)	19.0 (19.7)	6.9 (7.0)
Completeness (%)	90.65 (39.00)	90.48 (33.33)	99.9 (98.5)	99.91 (100.00)	99.56 (99.13)	98.56 (98.96)
Mean *I*/σ(*I*)	6.02 (1.12)	4.67 (0.41)	8.8 (0.3)	27.46 (2.95)	25.28 (2.54)	10.80 (2.38)
Wilson *B* factor (Å^2^)	118.66	24.16	29.03	62.84	27.12	35.43
*R*_merge_	0.3371 (3.593)	0.1787 (2.574)	0.120 (3.669)	0.1169 (1.255)	0.05639 (0.9895)	0.08972 (0.8622)
*R*_meas_	0.3611 (3.838)	0.1884 (2.709)	0.126 (3.879)	0.1187 (1.275)	0.05799 (1.0150)	0.09702 (0.9318)
*R*_p.i.m._	0.1272 (1.331)	0.05901 (0.8398)	0.040 (1.241)	0.0202 (0.2203)	0.01339 (0.2253)	0.03650 (0.3496)
CC_1/2_	0.990 (0.283)	0.998 (0.607)	0.999 (0.092)	1 (0.853)	0.999 (0.939)	0.999 (0.849)
CC*	0.998 (0.664)	1 (0.869)	1 (0.410)	1 (0.96)	1 (0.984)	1 (0.958)
Reflections used in refinement	12056 (516)	59954 (2186)	22075 (2193)	8708 (863)	38073 (3762)	16390 (1620)
Reflections used for *R*_free_	595 (24)	3116 (125)	1092 (54)	400 (45)	1951 (204)	782 (77)
*R*_work_	0.2823 (0.3762)	0.2375 (0.3108)	0.2347 (0.2983)	0.2021 (0.2901)	0.1820 (0.2585)	0.2172 (0.3178)
*R*_free_	0.3145 (0.3550)	0.2663 (0.3346)	0.2827 (0.3800)	0.2298 (0.2825)	0.2042 (0.2878)	0.2552 (0.3522)
CC(work)	0.468 (0.018)	0.827 (0.753)	0.799 (0.658)	0.955 (0.753)	0.963 (0.923)	0.957 (0.873)
CC(free)	0.471 (0.132)	0.781 (0.691)	0.673 (0.519)	0.919 (0.769)	0.962 (0.899)	0.935 (0.777)
No. of non-H atoms						
Total	4021	6714	5187	1920	2119	2606
Macromolecules	3926	5881	9828	1868	1960	2505
Ligands	91	6	0	20	22	4
Solvent	4	827	22	32	144	97
Protein residues	567	761	716	255	255	354
R.m.s.d., bond lengths (Å)	0.002	0.004	0.003	0.004	0.006	0.002
R.m.s.d., angles (°)	0.36	0.66	0.59	0.57	0.76	0.47
Ramachandran favoured (%)	92.74	96.56	95.38	96.81	97.21	96.86
Ramachandran allowed (%)	6.53	3.44	4.62	3.19	2.79	3.14
Ramachandran outliers (%)	0.73	0.00	0.00	0.00	0.00	0.00
Rotamer outliers (%)	0.32	0.17	0.22	0.00	0.00	0.00
Clashscore	4.61	5.77	1.22	5.83	2.57	1.88
Average *B* factor (Å^2^)						
Overall	121.67	29.15	35.13	65.07	41.57	52.33
Macromolecules	121.54	28.53	38.39	64.95	40.93	52.38
Ligands	128.82	48.85	N/A	82.66	69.49	56.14
Solvent	82.01	33.39	28.47	60.94	47.30	50.68
No. of TLS groups	10	10	16	2	0	12
